# Morphological Study before and after Thermal Treatment of Polymer-Polymer Mixed-Matrix Membranes for Gas Separations

**DOI:** 10.3390/polym16101397

**Published:** 2024-05-14

**Authors:** Pedro Pradanos, Cenit Soto, Francisco Javier Carmona, Ángel E. Lozano, Antonio Hernández, Laura Palacio

**Affiliations:** 1 Surfaces and Porous Materials (SMAP), Associated Research Unit to CSIC, Faculty of Science, University of Valladolid, Paseo Belén 7, 47011 Valladolid, Spain; marveliacenit.soto@uva.es (C.S.); fcojavier.carmona@uva.es (F.J.C.); lozano@ictp.csic.es (Á.E.L.); antonio.hernandez@uva.es (A.H.); laura.palacio@uva.es (L.P.); 2 Institute of Sustainable Processes (ISP), Dr. Mergelina s/n, 47011 Valladolid, Spain; 3 Institute for Polymer Science and Technology (ICTP-CSIC), Juan de la Cierva 3, 28006 Madrid, Spain; 4 IU CINQUIMA (Centro de Innovación en Química y Materiales Avanzados), University of Valladolid, Paseo Belén 5, 47011 Valladolid, Spain

**Keywords:** mixed-matrix membranes, gas separation, atomic force microscopy, etching with Ar plasma, grinding by polishing, thermal rearrangement, internal morphological characterization

## Abstract

A good integration of the polymer materials that form a mixed-matrix membrane (MMM) for gas separation is essential to reaching interesting permselective properties. In this work, a porous polymer network (PPN), obtained by combining triptycene and trifluoroacetophenone, has been used as a filler, which was blended with two o-hydroxypolyamides (HPAs) that act as polymer matrices. These polymer matrices have been thermally treated to induce a thermal rearrangement (TR) of the HPAs to polybenzoxazoles (β-TR-PBOs) through a solid-state reaction. For its structural study, various techniques have been proposed that allow us to undertake a morphological investigation into the integration of these materials. To access the internal structure of the MMMs, three different methods were used: a polishing process for the material surface, the partial dissolution of the polymer matrix, or argon plasma etching. The argon plasma technique has not only revealed its potential to visualize the internal structure of these materials; it has also been proven to allow for the transformation of their permselective properties. Force modulation and phase contrast in lift-mode techniques, along with the topographic images obtained via the tapping mode using a scanning probe microscope (SPM), have allowed us to study the distribution of the filler particles and the interaction of the polymer and the filler. The morphological information obtained via SPM, along with that of other more commonly used techniques (SEM, TGA, DSC, FTIR, WASX, gas adsorption, and permeability measurements), has allowed us to postulate the most probable structural configuration in this type of system.

## 1. Introduction

Polymeric membranes play a key role in gas separation processes with membranes. In fact, from a commercial point of view, they have taken over most of the market [1]. This is mainly due to a set of favorable properties, both economic and technological [2]. Their good permeability/selectivity ratio, as well as their acceptable mechanical and thermal properties in terms of ease of processing, make polymeric membranes one of the main materials for gas separation [3].

In 1991 and 2008, Robeson published two works setting the semi-empirical limit of the permeability–selectivity relationship for the simplest gas couples of industrial interest from a wide compilation of polymeric membranes [4,5]. Currently, most gas separation membranes made with the new-generation polymers are close to, or slightly exceed, the 2008 limit. Only in some cases is this limit undoubtedly exceeded, such as is the case of some thermally rearranged (TR) polymers [6,7,8,9,10,11] or polymers of intrinsic microporosity (PIM) [12].

However, both PIMs and TRs present some additional difficulties. In the case of PIMs, the large free volume they possess is lost over time due to the relaxation of the polymer chains. This process, known as *physical aging*, causes the membrane to drastically reduce its permeation properties [13]. The TR membranes are much more stable concerning their permeation properties than the PIMs ones, but they usually present worse mechanical properties than the starting polymers in membrane manufacture. With this type of system, a thermal treatment must be performed after the membrane molding process since TRs are insoluble [14].

Mixed-matrix membranes (MMMs) represent a way to improve the permeation properties of polymer-based membranes, allowing the Robeson limit to be exceeded with some ease [15]. These consist of the dispersion of a filler (organic, inorganic, or mixed) within a polymer matrix. In this way, by combining the properties of both materials, the selectivity and/or the permeability of these new membranes can be improved by taking advantage of the properties of the two materials and the synergy between them. This requires having fillers with very good permselective properties. If fillers with micropores are used, the free volume of the membrane increases, multiplying the possibilities for improving the properties of the MMMs. The most-used fillers are carbon molecular sieves (CMS), zeolites, ceramic materials, graphene, carbon nanotubes (CNT), or fullerenes in the case of inorganic fillers, and porous organic polymers/porous polymer networks (POPs/PPNs), conjugated microporous polymers (CMPs), metal organic frameworks (MOFs), or covalent organic frameworks (COFs) in the case of polymeric ones [16]. Within this set of fillers, crystalline molecular sieves (zeolites and MOFs) or amorphous ones (CMS and ceramic materials) are of additional interest due to their high entropic selectivity that allows for better discrimination between gaseous molecules of similar sizes [17,18,19]. This is mainly due to the rigidity of the porous structure [20]. However, obtaining a good MMM requires the overcoming of a series of compatibility problems. Among these are achieving good dispersion of the filler within the matrix preventing the rigidification of the polymer in contact with the filler or the appearance of free spaces at the interfaces. This is important since the functionality of an MMM is not only dependent on the individual properties of the components that form the material but also on their interaction and the resulting matrix structure after manufacturing [21].

To improve the compatibility between both phases, multiple solutions have been studied. For specific systems, the dispersion of components can be improved with ultrasonic stirring, high agitation, modifying the viscosity, charge, and solvents, by manufacturing the fillers in situ, etc. [19]. In general, organic fillers tend to have very good compatibility with the polymer matrix, improving adhesion and avoiding rigidification [22]. In the case of inorganic fillers, to reduce this problem, organic coatings of the filler particles have been used. Much work is currently being conducted in this domain, aimed at assembling membranes from customized building blocks. However, in most cases, the high cost of large-scale manufacturing hinders their industrial application [23,24].

However, results that appear in the literature on MMMs show that in some cases, their permselective properties are worse than those of their constituents. Moore and Koros, in 2005, studied the example of a polyetherimide filled with a zeolite. Their results revealed that the permeation of the MMMs obtained varied significantly depending on the integration of the two materials. Furthermore, the gas transport model through the matrix should be modified depending on the morphology acquired by the two phases and on the interaction between them [21]. In fact, several good flux models have been developed that predict the behavior of these systems as a function of their morphological characteristics [9].

Therefore, it is essential to establish an experimental procedure by which to study the morphology of MMMs once formed. The most common direct method to determine the existence of good compatibility between the constituents of MMMs is study using SEM images of cross-sections of the membrane [25]. Fair compatibility would lead to a good adhesion between the materials and to the absence of free spaces at the interfaces. However, this technique by itself is not decisive since in many cases, it is difficult to quantify these morphologies in enough detail. The determination of the fractional free volume (FFV) or the density of the MMMs compared to their constituents also informs us about free spaces or the penetration of the polymer into the filler matrix [26,27]. On the other hand, the rigidification of the polymer chains at the interfaces can be studied by analyzing properties such as the glass transition temperature (Tg) or the most probable intersegmental distance as a function of the filler content [14]. It is evident that, starting from a filler (normally porous) with good properties (permeability and selectivity), our aim should be to obtain a membrane with dispersed isolated particles, well integrated with the matrix (without existence of spaces at the interfaces), that does not stiffen the polymer and does not allow it to penetrate through the filler. Furthermore, for the same mass ratios, the smaller the size of the filler particles, the greater the interaction surface, leading to MMMs with better permselective properties [28]. This is because the selective surface flow is the relevant transport mechanism for condensable gases in MMMs with porous fillers [29].

In this work, various techniques were used to study the internal structure of the MMMs with a polymeric porous filler. These techniques include SEM, CO_2_ adsorption isotherms, permeation, AFM, etc. To carry out a complete and adequate internal structure characterization study, some techniques required careful prior processes, such as partial dissolution, polishing of the membranes, and treatment with argon plasma. At the same time, some techniques require several modes of application, so their complementarity is what allows use to obtain particular details that would not be shown otherwise. For example, the samples have been studied with AFM in tapping mode using both force modulation and phase contrast. This will allow us to obtain the size distribution of both the filler agglomerates and the particles that form these agglomerates.

The AFM study, together with complementary techniques, allows for the analysis of adhesion, rigidification, and the presence of gaps between the constituent phases of the MMMs. Morphological changes due to plasma treatment and their effects on the functional properties have also been analyzed. In addition to clarifying the structure of the MMMs, this allows us to observe the appearance of surface structures in the form of a network and to reveal modifications in the permeation properties of this type of system. The morphology of nanospheres that constitute the polymer matrix has also been studied.

The objective of the work, showing the observations obtained from all these techniques, procedures, and modes, is to capture the complementarity and need of all of them to determine the internal structure of the MMMs with a polymeric porous filler where the continuous phase is a polymer susceptible to suffering a TR process to another polymer structure once the MMM has been formed. A further objective of this work is to show the correlations between manufactured materials and morphology; this is shown throughout the work and in the Supplementary Materials.

## 2. Experimental Section

### 2.1. Materials

For the MMMs manufacture, a porous polymer network (which we will call here PPN2) has been used as a filler of two o-hydroxypolyamides (HPAs) that act as polymer matrices. These polymer matrices have been created through the reaction of the diamine 2,2-bis(3-amino-4-hydroxyphenyl)-hexafluoropropane (APAF) with two diacid chlorides: 2,2′-bis(4-chlorocarbonylphenyl)hexafluoropropane diacid chloride (6FCl) and 5′-terbutil-m-terfenilo-3,3″-dicarboxylic acid dichloride (tBTmCl). These HPAs, which we will call 6FCL-APAF and tBTmCl-APAF, have been thermally treated to induce a thermal rearrangement process (TR), transforming them from HPAs to polybenzoxazoles (β-TR-PBO) through a solid-state condensation reaction to obtain the polymer matrices TR-6FCL-APAF and TR-tBTmCl-APAF [14,27].

The synthesis of monomers, polymers, and PPN used in this work was previously described by the authors [14,27]. These works also describe the thermal rearrangement process as well as the preparation of the polymer matrix films and the MMMs (with schemes of both polymer and filler synthesis). However, because the manufacturing methodology could affect the PPN distribution within the polymeric matrix, a summary of the MMMs production process can be found in the Supplementary Materials (Section S1 of Supplementary Materials). The analysis of these correlations between manufactured materials and morphology is one of the objectives of this work. The choice of PPN as a filler may not seem very appropriate since it is difficult to distinguish the polymeric matrix with SEM [14]. But since this filler and the matrix have different viscoelastic properties, they can be differentiated with AFM methods.

Table 1 summarizes the nomenclature that we will use throughout this paper for each of the MMMs studied. The “#%” symbol indicates the weight percentage of PPN2 in the MMM. There is no tBtr0% sample because it was impossible to obtain this MMM free of defects (bubbles or pinholes) to allow them to be used as membranes for gas separation.

Because there is a morphological asymmetry of the samples when considering the face in contact with air (face A), in comparison with that contacting the glass (face B) in the casting process, we will mention the face on which the analysis is carried out to complement the nomenclature in Table 1.

The membranes and polymers involved in this work have been previously characterized in multiple aspects by the authors in the context of MMMs for gas permeation [14,27]. Therefore, in the following sections, only the techniques used specifically for this work are described.

### 2.2. Polymer Characterization

Weight-average molecular weights (Mw) and number-average molecular weights (Mn) of the polymers synthesized were determined via gel permeation chromatography (GPC) using a Tosoh Ecosec HLC-8320GPC (Tosoh, Tokyo, Japan) device. Samples were prepared by dissolving 0.5 mg of each polymer in 2 mL of THF and filtered through a 0.45 µm filter.

### 2.3. TGA, DSC, FTIR, WAXS, SEM, CO_2_ Adsorption Isotherm, and Gas Permeability

To analyze the effects of argon plasma treatment on the membranes, several techniques have been used:

1. The thermogravimetric analysis (TGA) was performed using a Q500 thermogravimetric analyzer from TA Instruments (New Castle, DE, USA). A heating ramp mode from room temperature up to 800 °C at a rate of 10 °C/min was used with samples of around 5 mg under a 50 cm^3^/min flow rate of ultra-high-purity nitrogen and a flow rate of 40 cm^3^/min through the scale chamber.
2. The glass transition temperature values were determined with a Differential Scanning Calorimetric (DSC) DSC-25 Analyzer (also from TA Instruments). DSC analyses for TR polymers were carried out at a heating rate of 20 °C /min up to 360 °C. In all cases, the experiments were performed under an ultra-high-purity N_2_ atmosphere using 6–10 mg of membrane in gas-tight aluminum containers. The *T_g_* was determined in the first heating cycle from the inflection point (*T_i,g_*) of the measured DSC curve in the glass transition region.
3. Attenuated Total Reflectance-Fourier Transform Infrared (ATR-FTIR) spectra were performed using a Perkin Elmer Spectrum One FT-IR (Perkin-Elmer, Waltham, MA, USA) coupled with a universal attenuated total reflection (ATR) sampling module with a diamond-tipped probe, following the band’s intensity.
4. The membranes were also tested via wide-angle X-ray scattering (WAXS) at room temperature using a Bruker (Bruker, Billerica, MA, USA) D8 discover A25 advanced diffractometer equipped with a Goebel mirror. The LynxEye detector was operated at a speed of 0.5 s with a step scanning mode ranging from 5° to 70° and a 2θ step of 0.020°. A Cu Kα (λ = 1.542 Å) radiation source in a ceramic tube was used.
5. The adsorption isotherms were carried out in a volumetric device Autosorb IQ (Quantachrome Instruments, Boynton Beach, FL, USA). The samples were first measured for CO_2_ at 273.15 K (up to $p/p_{0}=0.03$, afterwards with N_2_ at 77 K (up to $p/p_{0}=1$). Samples were degassed at 100 °C for 10 h under vacuum before the sorption measurements to eliminate possible adsorbed gases or water vapor. The adsorption isotherm data were used to obtain the pore size distribution via the non-local density functional theory equilibrium model (NLDFT). Acquisition and calculation were carried out by Quantachrome^®^ ASiQwin software (version 5.21).
6. Both permeability and ideal selectivity of the membranes have been determined by using a constant volume-variable pressure apparatus at 35 °C and an upstream pressure of 3 bar. The gas flow through the membrane is determined by measuring the pressure versus time on the low-pressure side when the system reaches steady-state conditions, as described elsewhere [14,27]. All the gases used have purity greater than 99,999.

The plasma-treated and untreated samples came from the same film to avoid dispersion due to the sample manufacturing process.

### 2.4. Procedures to Study the Integration of PPN Particles within the Polymer Matrix

To study the distribution of PPN2 particles inside the matrix, three procedures were used: surface polishing, partial solution, and surface etching with argon plasma. The sample characterization was carried out with SEM and AFM.

#### 2.4.1. Description of the Polishing Process and Device

The samples’ surface was roughened with a Beta Grinder–Polisher at a speed of 400 rpm and cooled with water. The samples are fixed with adhesive tape to a metal support that allows for the regulation of the thickness of the sample exposed to the blasting process. The process was carried out in three stages. In the first one, a Silicon Carbide grinding paper of 15.3 µm grit size (CarbiMet Plain 600 [P1200]) is used. In the second stage, a hard polishing cloth (TexMet P, PSA) was applied with a MetaDi monocrystalline diamond suspension of 3 µm. Finally, a soft polishing cloth (MicroCloth^®^, PSA) was used with Micropolish alumina suspension, containing agglomerated alumina particles of 0.05 µm size (Micropolish^®^ II). The polisher and all the required devices, such as sandpaper or particle suspensions, were obtained from Buehler (Lake Bluff, IL, USA).

#### 2.4.2. Description of the Partial Dissolution Process

A second set of samples from the same batch has been subjected to partial dissolution of the MMMs surfaces, using the same solvent as in the manufacturing process (N-methylpyrrolidone, NMP). NMP readily dissolves HPA, but this solvent does not solubilize TR membranes or PPN2 filler. For partial dissolution, a drop of NMP was deposited on the surface of the samples for 15 s, and there were subsequently washed with water to remove the NMP rests. The optimal time deposition for HPA samples was determined after several tests, in which it was found that longer times cause a substantial part of the HPA to disappear, with a large amount of PPN2 appearing on the surface. In the case of TR-MMMs, since they are insoluble in this solvent, the NMP drop was deposited for one hour before being washed with distilled water. In this process, the TR material was not dissolved, but the parts of the polymer that had not undergone the thermal rearrangement process were eliminated. In the TR-membranes, NMP has also been deposited on the samples that have previously undergone the complete polishing process to remove impurities from the surface.

#### 2.4.3. Surface Etching with Argon Plasma Process

Plasma etching was performed in a radiofrequency plasma chamber (Expanded Plasma Cleaner PDC-001 from Harrick Plasma (Ithaca, NY, USA)) connected to a flow meter (PlasmaFlo PDC-FMG, Harrick Plasma, Ithaca, NY, USA) and a vacuum pump. An argon flow of 0.50 cm^3^/min STP at a 75 Pa of pressure was used. The system allows for operation at three powers: 7.2, 10.2, and 29.6 W (8–12 MHz). To analyze the effects of plasma, samples were treated at different times and using different powers. Table S1 in the Supplementary Materials shows the technical data. Using an inert gas leads to a simple elimination of the surface layers (etching) of the samples, without any deposition. However, the formation of radicals can cause additional cross-linking of the polymer chains. By controlling the time and the power of the system, it is possible to obtain images at different depths of the polymeric matrix, although it has been proven that for times over 8 h at maximum power, significant differences are no longer detected in the topography of the samples. In turn, for all samples, it is observed that a short treatment with plasma at low power eliminates the layer of contamination deposited on the surface and increases the resolution of the images, while more aggressive treatments remove some polymer from the surface of the samples.

#### 2.4.4. Scanning Electron Microscopy (SEM)

A scanning electron microscope, QUANTA 200 FEG ESEM (Thermo Fisher Scientific, Waltham, MA, USA) was used with a voltage of 1.5 kV in a high vacuum and using the secondary electron detection method. The samples were metallized with gold to increase the resolution of the images. In addition to surface imaging, samples were cryogenically fractured using liquid nitrogen to obtain information from the cross-section images. All samples were analyzed both before and after plasma treatment.

#### 2.4.5. Atomic Force Microscopy (AFM)

The atomic force microscope used was a Nanoscope Multimode IIIa from Digital Instruments (Veeco Metrology Inc., Santa Barbara, CA, USA). All the measurements were made in tapping mode to avoid damaging the samples. Both topographic and phase contrast images were collected simultaneously. These last ones inform us of changes in the cantilever oscillation phase due to changes in the viscoelastic properties of the surface or to abrupt changes in the topography. According to the tips manufacturer’s specification (Veeco Metrology Inc., Santa Barbara, CA, USA), the cantilever length is 140–180 µm, the angle of attack is lower than 10°, the radius of curvature of the conical tip lower than 5 nm, and the oscillation frequency ranges from 296 to 351 kHz (~12 × 10^−3^ N/m) [30].

The phase contrast images obtained do not allow us to differentiate the distribution of the different materials that make up the surface due to the interference of the topographic changes. To avoid this drawback, measurements were carried out via the *interleave scanning* technique in *negative-lift* mode to acquire the phase images. In this way, during the scanning of the surface, the tip first runs over the surface to acquire its topography and then scans the surface at a constant height and the system records the phase signal. In this way, all interferences from the topography are minimized [30].

Finally, to magnify the differences between surface materials, the force modulation mode has been used along with interleave scanning and negative lift. In this case, a cantilever with a lower elastic constant was used, choosing the elastic constant value depending on the material to be detected. The cantilever dimensions are 225 × 27 × 2.7 µm, and its natural frequency is between 43 and 81 kHz, with an elastic constant in the range of 0.6–37 N/m according to the manufacturer’s specifications (AppNano, Mountain View, CA, USA). The radius of the conical tip is less than 10 nm, and the length of the tip is between 14 and 16 μm. Using this method, which is similar to that previously described, two scans were conducted. In the first one, at the natural frequency of the tip, the topography is recorded. In the second run, at a constant height, the oscillation amplitude signal is measured when the tip is made to oscillate at the natural frequency of the piezoelectric material (7.03 kHz in our case) that generates the oscillation, which is much smaller than the natural one of the cantilevers. In this measurement, the tip hits the surface and produces a rebound of the cantilever whose amplitude depends on the elasticity of the surface. This contributes to differentiating materials with different viscoelastic properties.

In our case, three channels were recorded simultaneously: topography (tapping mode), phase (tapping mode with negative lift), and amplitude (force modulation). Depending on the sample type, the height values in a negative lift for force modulation were between −1 and −10 nm. Due to the lower oscillation frequency and the large curvature radius, the resolution of the topographic and phase images is significantly lower than for normal-tapping mode. Resolution in *x–y* is sacrificed to be able to quantify the viscoelasticity of the material measured in the *z*-direction. This type of analysis (tapping height, tapping phase, tapping negative-lift phase, and tapping negative-lift force modulation) was carried out with polished samples, partially dissolved, and treated with argon plasma at different times and powers (see Table S1). The image analysis was conducted with three different software programs: NanosScope Software V4.23 from Digital Instruments, NanoScope Analysis Software 32 1.70 from Bruker, and the free image-analysis software, ImageJ V1.51j8 [31].

## 3. Results and Discussion

### 3.1. AFM Characterization

#### 3.1.1. Polished Samples

As described in Section 2.4, the samples are first grinded by a few microns (~10–20 μm) so that their interior emerges to the surface, and second, they are subjected to two sequential polishing processes. The latter allow for the obtaining of a smooth surface that prevents topographic irregularities from masking the information. The grinding and polishing processes were carried out on both sides of the membrane to analyze if there are differences as a consequence of the effects of gravity and surface tension in the manufacturing process of MMMs (see Section 2.1 and Supplementary Materials).

**Figure 1:**
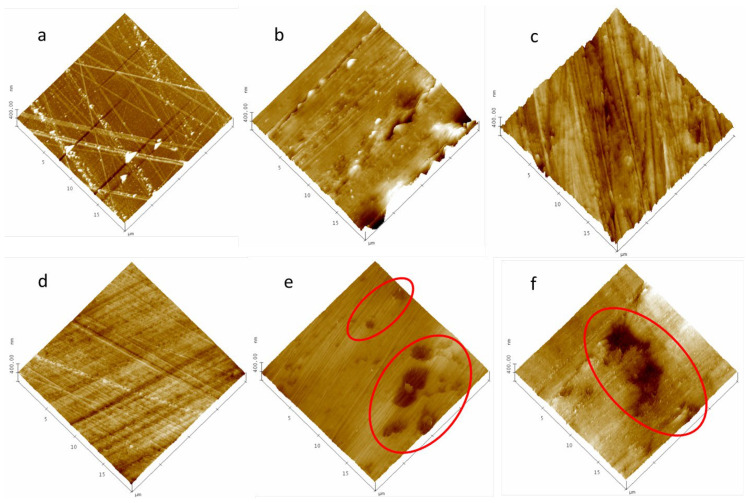
Simple 6F0% (**a**–**d**): without grinding treatment (**a**); griding paper 15 µm (**b**); hard polishing cloth + diamond suspension 3 µm (**c**); soft polishing cloth + alumina suspension 0.05 µm (**d**); 6F10% with all polishing treatments (**e**); 6Ftr10% with all polishing treatments (**f**).

In Figure 1a–d, a series of images of face A for 6F0% samples show how the surface topography changes from the no-grinding state (Figure 1a) to the highest polishing degree (Figure 1d). In this last case, the surface is very homogeneous, and only the grooves caused by the alumina particles used in the last phase of polishing can be appreciated. However, when this process is conducted on samples with PPN2, a series of holes are observed. The population density of these holes is proportional to the PPN2 content in the MMM. In Figure 1e,f, two examples of 10% MMMs side A are shown for non-TR membranes (6F10%) and TR ones (6Ftr10%), respectively. According to these figures, the holes (marked with red circles in the figure) are detected regardless of the thermal treatment (375 °C for the TR membranes) undergone by the MMMs.

To determine the proportion of the surface with voids versus the flat surface, a statistical bearing analysis was performed on different samples (Figure S1 shows an example of this analysis).

To obtain values with statistical significance, this analysis was carried out on 20 × 20 µm images taken at five different points of the samples, both for faces A and B, observing no significant differences between the two sides within the range of error of the method. Therefore, each MMM was assigned a percentage of holes calculated as the average of 10 images of both sides.

**Figure 2:**
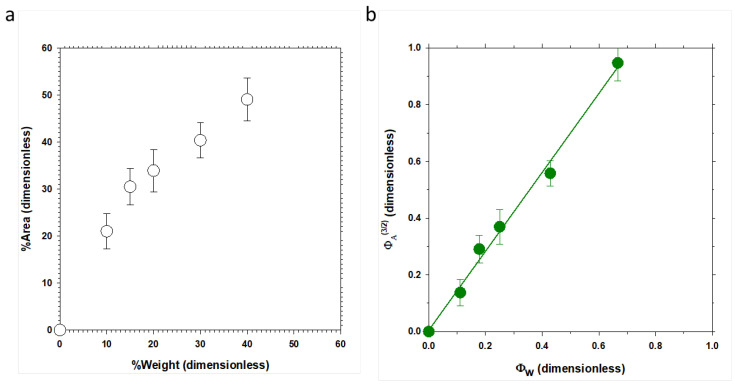
Percentage of surface corresponding to holes versus % *w*/*w* of PPN2 (**a**) and area fraction $(\Phi_{A}^{3/2}$) versus weight fraction ($\Phi_{w}$) (see the text for the precise definitions of $\Phi_{A}^{3/2}$ and $\Phi_{w}$) (**b**) for the series 6F#%.

Figure 2a shows an example of how the percentage of surface corresponding to holes varies versus the % *w*/*w* of PPN2 in the 6F#% membrane series. Similar behavior is observed for the series 6Ftr#%, tB#%, and tBtr#% (Figure S2 shows another example).

**Figure 3:**
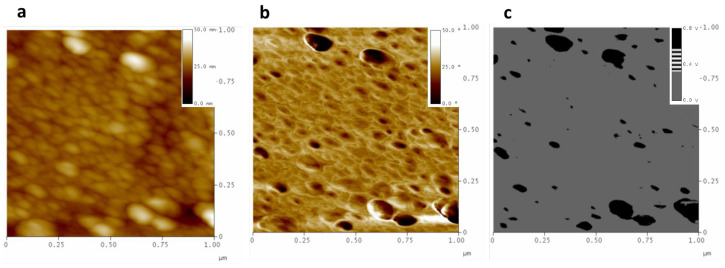
MMM 6F10% polished images: topography (**a**); phase contrast (**b**); amplitude obtained with the interleave scanning technique in negative-lift mode in FM (FM-Map) (**c**).

From Figure 2a, it can be deduced that there is no linear correlation passing through the origin and data are above the bisector. This is to be expected since, among other factors, we are relating area percentage and weight percentage, and we must consider geometric and density relationships to be able to compare both magnitudes. If we assume an MMM as a distribution of spherical particles (filler) in the polymer matrix, the area fraction of the two components can be related to their volumes by the following:

(1) $$\frac{V_{filler}}{V_{matrix}}={\left(\frac{A_{filler}}{A_{mztrix}}\right)}^{3/2},$$

where $V_{filler}$ and $V_{matrix}$ are the volumes of PPN2 and the polymer, respectively, and $A_{filler}$ and $A_{matrix}$ are the corresponding areas for a random cut in the MMM. The weight fraction (Φ_w_) of the filler can be evaluated as follows:

(2) $$\Phi_{w}=\frac{W_{filler}}{W_{matrix}}=\frac{\rho_{filler}V_{filler}}{\rho_{matrix}V_{matrix}}=\frac{\rho_{filler}}{\rho_{matrix}}{\left(\frac{A_{filler}}{A_{matrix}}\right)}^{3/2}=\frac{\rho_{filler}}{\rho_{matrix}}{\left(\Phi_{A}\right)}^{3/2},$$

with $W_{filler}$ and $W_{matrix}$ being the weight of each component in the MMM, and $\rho_{filler}$ and $\rho_{matrix}$ being their respective densities (included in Table 2). This behavior can be detected in Figure 3b, where the slope must correspond to the density relation, if we suppose an ideal mixture (without holes) and no interaction.

The density of PPN2 is $\rho_{filler}=0.991$ g/cm^3^ [27]. Comparing the third and fourth columns of Table 2, it seems that the value for the $\rho_{matrix}$ obtained by AFM is between 5 and 10% lower, which would indicate that there are PPN2 particles (or holes left by them) not detected by this method. This method is unable to detect PPN2 particles or interstices below 100 nm. It is worth noting that the presence of bubbles or interstices among the detected holes would lead to density ratios by AFM greater than those corresponding to experimental densities.

The distribution of holes in the MMMs with 10% and 15% of PPN2 was also studied. Figure S3 shows an example for the 6F10% sample, and the data of the mean value (μ) and its standard deviation (σ) are given in Table 3. For higher percentages, the high population produces the connection between the holes of the surface, making difficult the interpretation of the distributions. For these percentages, results indicate that the agglomerates have sizes between 2 and 3 μm.

As seems logical, the grinding and polishing processes increase roughness ($R_{q}$). After the last polishing step with alumina suspension, roughness returns to the original values. At the same time, this last step allows us to see the “*craters*” left by the extraction of the filler agglomerates. As expected, a clear increase in $R_{q}$ is also observed with the filler content (the greater the number of holes on the surface, the greater the roughness). A more detailed analysis of the evolution of $R_{q}$ is included in the Supplementary Materials (Figure S4).

Although the presence of holes could be due to poor adhesion between the filler and the matrix, we were able to verify that it is rather due to the cohesion of the PPN2 particles forming agglomerates. To confirm this, the results from phase contrast signal (tapping mode) (Figure S5) and the interleave scanning technique in negative-lift mode were analyzed. Both techniques demonstrated that the phase changes were mainly due to changes in the viscoelastic properties of the different materials and not due to topographic changes (Figures S6 and S7; Table S2). The results showed the existence of a high density of PPN2 particles adhering to the surface where the agglomerates were detached, confirming the good adhesion of the filler with the polymer (Figure S7 and Table S2). As a last check to verify the areas of different hardness (see Section 2.4), the interleave scanning technique in negative-lift mode was carried out in force modulation (FM) (Figure 3).

In the topography and phase contrast images (Figure 3a,b), no differences are recognized between the filler and the polymer, while Figure 3c (which we will call a force modulation map (FM-Map)) clearly shows the PPN2 particles, supporting their greater hardness compared to the polymer, since the black tone represents a greater amplitude of cantilever oscillation than the polymer itself (grey tone). The FM images corroborate what was already seen before; i.e., that for the samples without filler particles (6F0% 6Ftr0%, tB0%, and tBtr0%), there are no differentiated areas; but in the samples with PPN2, a high population of particles has been found at the bottom of the holes produced by grinding. Referring now to the comparison of samples without and with TR (6F#% and 6Ftr#%), these show similar hardness in both polymers. This agrees with previous results that showed that the Young’s modulus values of both polymers are of the same order of magnitude (E~3.8 GPa for 6F0% and E~2.5 GPa for 6Ftr0%) [14]. However, the E values obtained by FM should be used with caution since they depend on several factors such as the radius of curvature of the tip, among others [32].

The FM-Map allowed for the analysis of the distribution of PPN2 particles embedded in the surface. Table 4 shows the Gaussian fitting parameters from the analysis of at least five FM-Map images, with 1 × 1 μm and 5 × 5 μm, using ImageJ. The values do not show significant differences from those derived from other AFM techniques (Table S2). In all cases, these are broad distributions with values centered between 70 and 87 nm, similar to those determined in previous work using SEM for the characterization of PPN2 [33].

#### 3.1.2. Partial Dissolved Samples with NMP

**Figure 4:**
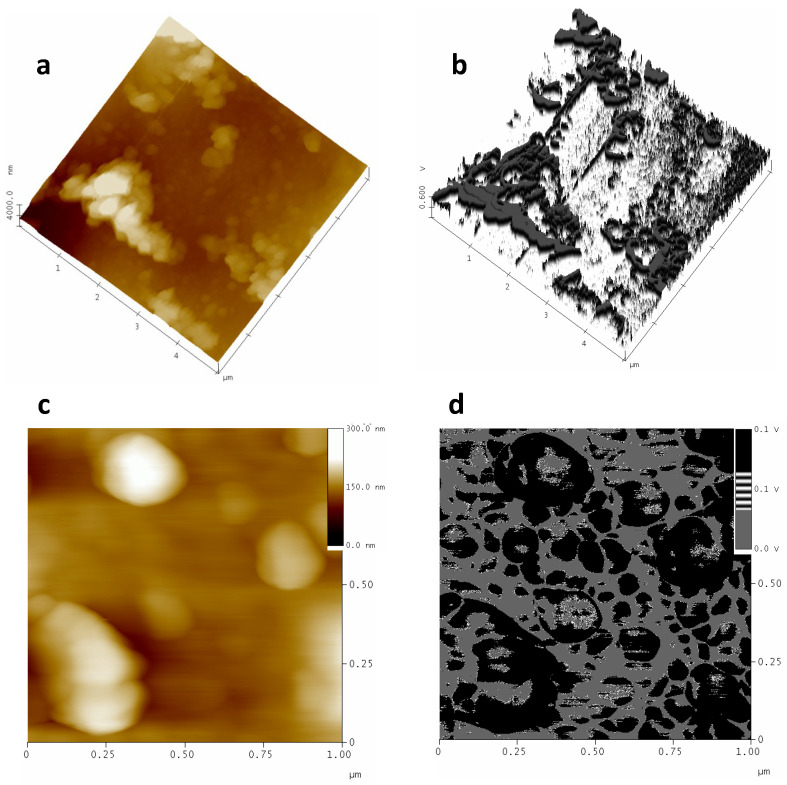
6F15% with its surface partially dissolved with NMP: topography images (**a**,**c**); FM-Map (**b**,**d**).

Treating the surface with a solvent that superficially removes the continuous phase without affecting the filler will provide information on the distribution of the PPN2 particles in the matrix. As mentioned in Section 2.4, the non-TR polymers are dissolved very easily by adding a drop of NMP to the surface. With a normal 10× microscope, the depression created is easily observed. This causes a large part of the polymer to be removed and the PPN2 particles to emerge, generating a surface with higher roughness, as shown in Figure 4a (note *z*-axis). Attending to $R_{q}$, it increases more than 20 times after partial dissolution (for 5 × 5 μm images: $R_{q}$ = 206 nm from Figure 4a; $R_{q}$ = 7.7 nm from Figure S4a). The FM image (Figure 4b) clearly shows that the accumulation of particles reached sizes close to those of the holes that appear after the grinding and polishing processes, around 2–3 μm (see Table 3). Some traces of embedded polymer between the PPN2 particles are also noticeable since the FM signal presents discontinuities concerning the topographical signal. Figure 4c,d show the topography and FM-Map for the same MMM at higher magnification, which is intended to enable us to detect the individual particles that make up the PPN2 agglomerates more clearly in the FM-Map. The particle size distribution of each sample shows very similar results for both sides, for the different proportions, and for the two polymers. However, the mean values and their standard deviations for all images studied (135 ± 98 nm) are slightly higher than those shown in Table 4 for polished samples. This increase in size can be attributed to the fact that the presence of polymer between PPN2 particles does not allow for the individualization of some of them.

#### 3.1.3. Plasma-Treated Samples

**Figure 5:**
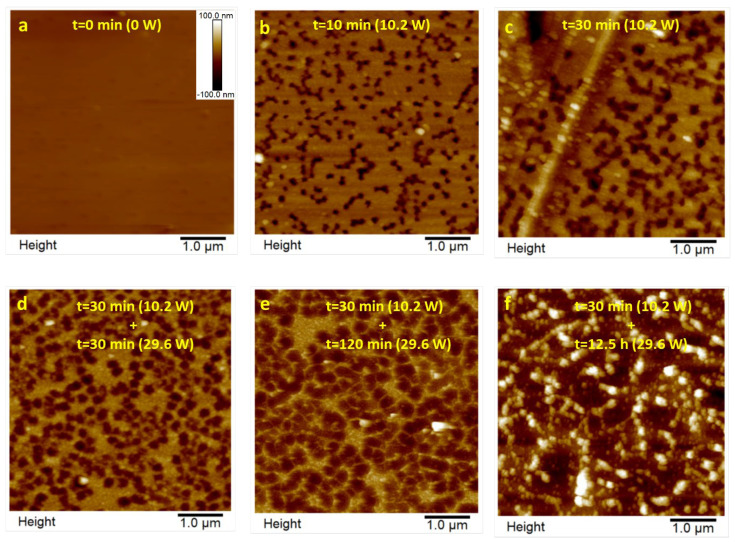
6F0% sample sequentially treated with Ar plasma (**a**–**f**). Times and power are indicated in the images.

Argon plasma was used as a third roughing-out method. Figure 5 shows the evolution of surface topography as a function of time and plasma power for the face A of the 6F0% membrane. After removal of the contamination layer (Figure 5a), in Figure 5b, we detect a population of 230 ± 45 nm pores that do not pass through the membrane (depth = 90 ± 27 nm). These dead-end pores have only been observed on the air-exposed side in the film formation process of the 6FCl-APAF membrane, lacking PPN2 and without thermal treatment (Figure S8). They can be attributed to solvent bubbles that are trapped on the top surface in the diffusion process during formation. The diffusion/surface tension conditions are different when it comes to the other polymer (tB0%) and when PPN2 is present. It seems clear that the thermal rearrangement causes these bubbles to disappear because they are not observed in 6Ftr0% either (Figure S8).

From the AFM analysis of the depth lines, it is possible to quantify how plasma etching modifies the depth (see an example in Figure S9). This modification not only affects the top layer that defines the dead-end pores but also its bottom (see compilation Table 5). It has been observed that for times greater than 8 h of plasma treatment at maximum power, the appearance of the surface is very similar to the image in Figure 5f.

**Figure 6:**
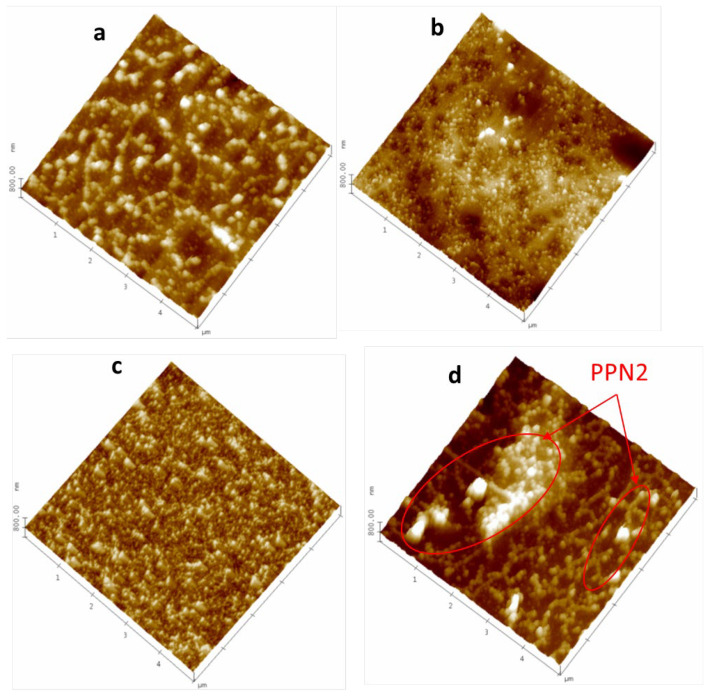
Samples treated with Ar plasma for 30 min at 10.2 W plus 12.5 h at 29.6 W. 6F0% (**a**), 6Ftr0% (**b**), tB0% (**c**), and 6F10% (**d**).

Figure 6 corresponds to samples after long plasma treatment. We notice that the framework observed in Figure 5f (sample 6F0%) is common for all the samples studied within the areas without PPN2. However, some differences are observed depending on the type of polymer (Figure 6a vs. Figure 6c) or if the polymer has undergone a TR process (Figure 6a vs. Figure 6b). Furthermore, we see that PPN2 particles have greater resistance to plasma attack, emerging in the images as projections on the surface (Figure 6d).

The study of both the particle size distribution and the surface area occupied by the agglomerates of PPN2 (for MMMs of 10%) gave results comparable to those obtained with the polished samples. In the case of samples treated with plasma, the mean value of the particle size distribution is a little lower (1.98 ± 1.74 μm versus 2.37 ± 1.26 μm) due to the increase in the population of smaller particles not detected in the polished samples. Possibly, the polishing process did not remove PPN2 particles which were isolated or forming small agglomerates; therefore, no holes were produced.

The study of the surface roughness of the AFM images shows that $R_{q}$ grows rapidly with the plasma treatment time, but after 180 min, the growth becomes moderate, and it also grows with the TR process and due to PPN2 presence, increasing with PPN2 percentage (Figures S10 and S11). For example, with 30% of PPN2, the increase in $R_{q}$ exceeds 400% to the initial roughness for long treatment times.

**Figure 7:**
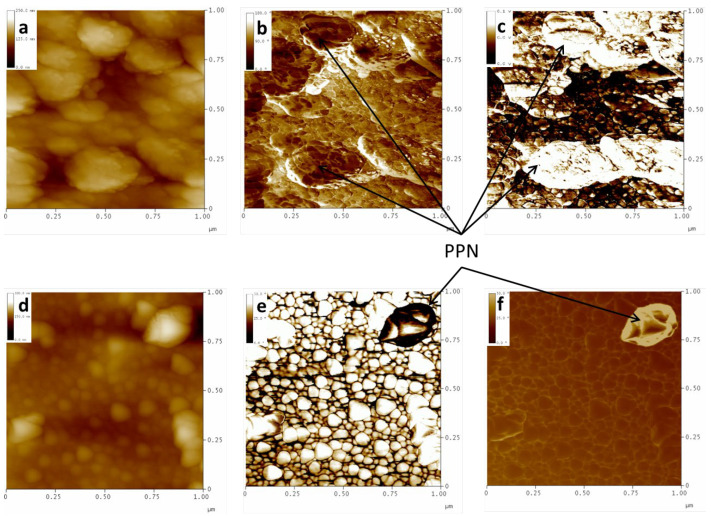
The 6F10% samples treated with Ar plasma for 30 min at 10.2 W plus 12.5 h at 29.6 W. Top row: images with FM tip (70 kHz) of topography (**a**); phase (**b**); FM-Map (**c**). Botton row: images with tapping mode tip (300 kHz) of topography (**d**); phase contrast (**e**); phase contrast by using negative lift (**f**).

To verify that these bumps are PPN2 and not simply some areas less etched by the plasma, we once again use the FM technique and the phase contrast images, along with the interleave scanning with negative lift. Figure 7 shows the presence of PPN2 using the FM tip (Figure 7a–c) and tapping-mode tip (Figure 7d–f). The FM method clearly shows that the lighter structures (higher voltage in the signal) in Figure 7c have higher hardness (PPN2) than the darker areas (polymer). Therefore, the phase changes observed in Figure 7b are not only due to changes in the topographic properties but also to changes in the viscoelastic properties of the surface. Furthermore, because the image shows areas with polymer, this proves that the plasma does not easily tear off the polymer in contact with the PPN2 surface; this is due, once again, to the good integration between PPN2 and the polymeric matrix.

The phase contrast with negative lift (an example in Figure 7f) shows the presence of a PPN2 particle with great definition. Although this particle, along with others, is clearly shown in the topographic image (Figure 7d) and phase contrast without lift (Figure 7e), it is the only one isolated by showing a differentiated signal in the image of Figure 7f, corroborating that it has different viscoelastic properties from the rest of the surface. The effects of topography are maintained in Figure 7e, since the polymer grains are very clearly defined by the topographic effects, which were minimized in the last image using negative lift with interleave.

A comparison of the topographic images obtained via both techniques (Figure 7a versus Figure 7d) demonstrates what was mentioned in Section 2.4 regarding the greater x–y resolution of the phase contrast technique with a higher frequency tip (and lower radius of curvature) than the frequency required to use force modulation.

**Figure 8:**
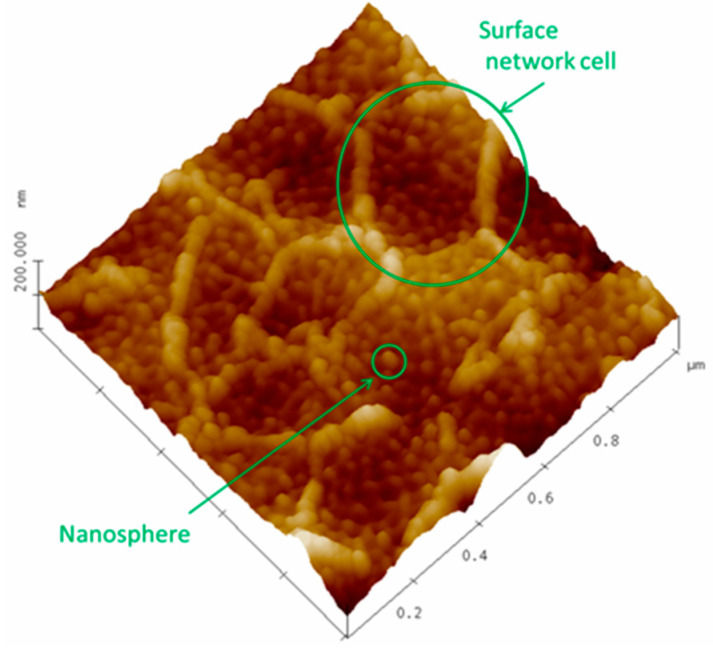
The 6F0% sample treated with Ar plasma for 30 min at 10.2 W plus 12.5 h at 29.6 W, highlighting both a nanosphere and a surface network cell.

The good resolution of the images after plasma treatment has allowed for an analysis of the sizes of the nanospheres defining the matrix polymer using topographic (Figure 8) and phase contrast images of 1 × 1 μm (Figure S12). The results of the statistical study of the size distribution of these nanospheres are shown in Table 6. For each of the polymers and their TR, the values of all the samples studied have been averaged because no significant statistical differences have been found between them. Similarly, Table 6 also shows the average size of the surface network cell generated by the plasma treatment for the same polymers shown in Figure 6 and highlighted as an example in Figure 8 (Figure S12).

These results show that the sizes of both the nanospheres and the surface network cells are mainly associated with the nature of the polymer.

Moreover, the thermal treatment does not substantially modify these structures. Regarding the nanospheres observed in all cases (untreated, plasma-treated surfaces, polished, or NMP-dissolved samples), they have a similar size for a specific polymer and its corresponding TR. According to this, we considered the convenience of an estimation of how many repeating units (chain links) these nanospheres form, as well as the number of polymer molecules. Data for the specific volume per repeating unit molecule (*V*) can be obtained from the density of the polymer and the molecular weight of the repeating units (see Table 2 and Table 7) [14,27]. Thus, with the volume of the nanospheres ($V_{ns}$), the number of repeating units in each nanosphere was calculated (${\langle N_{i}\rangle }_{ns}$) (Table 7).

(3) $${\langle N_{i}\rangle }_{ns}=\frac{V_{ns}}{V}$$

The molecular weight of the polymers without thermal treatment has been determined via gel permeation chromatography (GPC), while for the TRs, it has been calculated theoretically assuming that the transformation occurs at 100%. The results, along with the molecular weight of the repeating units (*M_i_*), are shown in Table 7.

With these data, the number of polymer molecules for each nanosphere can be evaluated as follows:

(4) $${\langle N_{i}\rangle }_{ns}=\frac{V_{ns}M_{i}}{M_{n}}.$$

Results for ${\langle N\rangle }_{ns}$ are also shown in Table 6. Important differences are detected between the two polymers, although not so much between the starting polymers and their TR. It is difficult to guess what causes polymers to opt for this type of packaging, although it surely has to do with the configurational properties of the polymer chains and with the polymer–solvent interactions in the evaporation process necessary to form the film. Even so, in analyzing the *M_n_* values, an important relationship between molecular weight and the number of molecules per nanosphere and their size can be seen. In any case, this type of structure, which shows the surface of a polymer formed by a packing of nanospheres (referred to as “nodules” or “nodule aggregates” depending on their size), has been widely observed by many authors [34,35,36,37].

As we have already mentioned, the average cell size of the reticular structures that appear as a consequence of the Ar plasma treatment may be associated with the nature of the polymer (see average values in Table 6) since this structure is preserved after the TR process and does not seem to be significantly altered by the presence of PPN. It could be thought that these lattices are caused by the plasma itself since the typical size of the Debye length of an Ar plasma under the conditions of this work ranges between 50 μm and 400 μm [38], i.e., values that are compatible with the observed cell sizes in AFM images. However, the images demonstrate that this network has different viscoelastic properties than the matrix that supports it (see Figure S13).

### 3.2. Other Characterization Technique

#### 3.2.1. ATR-FTIR and WAXS Measurements

To analyze whether the plasma modifies the properties of the polymers and PPN2 in the MMMs, TGA, DSC, ATR-FTIR, and WAXS measurements, thickness measurements, adsorption of CO_2_, and gas permeability were carried out before and after treatment with Ar plasma for a time of 8.5 h (510 min) at 29.6 W.

The FTIR spectra do not show significant variations. This seems to indicate that there are no important modifications of the functional groups of the polymer or PPN2 (some examples are shown in the Supplementary Materials, Figure S14). In any case, if the plasma modifies a layer of a few nm on the surface (either functionally or by cross-linking), this could not be detected in the FTIR spectra due to the greater penetration depth of the infrared radiation beam (0.5–5 µm [39]). The same occurs with the TGA analyses; the behavior is identical in both cases for all the membranes studied in this work (an example is shown in the Supplementary Materials, Figure S15). Therefore, the plasma treatment does not affect the TR process (see Figure S16). The thickness measurement also does not show significant differences after the maximum plasma treatment. In all cases, the difference found is lower than the experimental error (approximately 5 µm).

For WAXS measurements, a decrease in the average value of the intersegmental distance of 2.5% is observed after plasma treatment only in the case of MMMs with the tBTmCl-APAF polymer.

#### 3.2.2. Gas Permeability Characterization

For permeability measurements, three gases have been chosen (He, N_2_, and O_2_) that, in addition to having important industrial applications (He/N_2_ and O_2_/N_2_ separations for example), present low interaction with the polymer matrix. In this way, we prevent the plasticization effects that could be produced by gases such as CO_2_ or CH_4_ and could mask the effects of the Ar plasma treatment. Differences were observed between the untreated and treated samples. In the case of membranes with pure polymer, a decrease of 25% in permeability is observed for tB0%, and a decrease of 10% is observed for 6F0% (values averaged for the three gases). However, in the case of the MMMs of both polymers, permeability increases between 5% and 30%, with a large margin of error, depending on the sample and without clear trends. The randomness of the presence of large agglomerates of PPN2 particles or the lack of spatial homogeneity of the plasma may be the cause of the low reproducibility of permeability. Variations in selectivity are not significant within the error margin.

**Figure 9:**
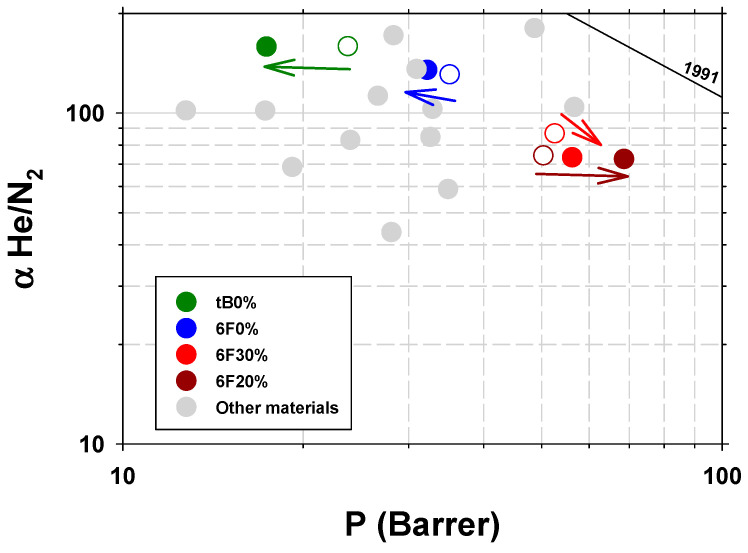
He/N_2_ selectivity versus He permeability for the studied samples. Void circles correspond to the plasma-untreated membranes and the filled circles to the samples treated with Ar plasma for 8.5 h at 29.6 W. Other literature data have been included [40].

**Figure 10:**
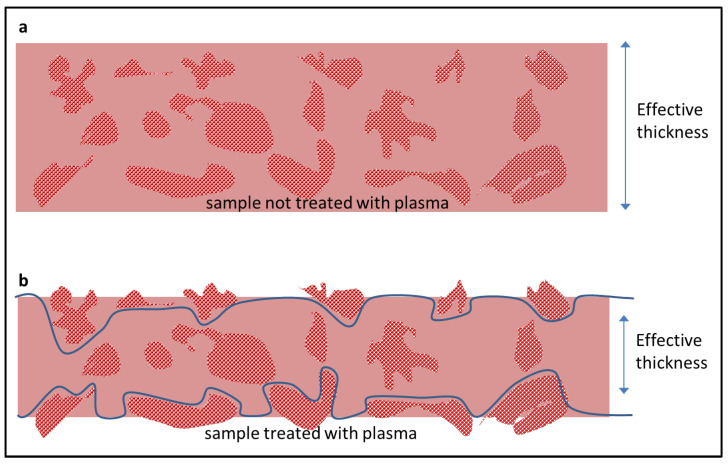
Scheme of the cross-section of an MMM showing the change in the effective thickness for the permeation before (**a**) and after (**b**) the plasma treatment.

Examples are shown in Figure 9, in a Robeson-type diagram for the He/N_2_ [4]. It is seen how the plasma treatment produces a decrease in He permeability for pure polymers and an increase in MMMs (trend indicated in Figure 9 by arrows of the same color as the samples). A possible explanation for the behavior of pure polymers is that the plasma treatment produces a more cross-linked polymer layer on the surface of the sample that acts as a supplementary barrier for diffusivity. This would explain why no differences are observed in the case of TGA and FTIR analyses. In the case of WAXS, intersegmental distances are detected at the smaller angles and therefore with the smaller beam penetrations, and even then, small decreases in the intersegmental distance are only observed when the decrease in permeability is significant (tB0%). It is also difficult to explain why MMMs increase their permeability with these gases due to plasma treatment. A possible cause may be the decrease in its effective thickness due to the presence of PPN2 agglomerates. As we see in Figure 6d, plasma treatment can erode the surface and expose an important portion of the PPN filler. These PPN inclusions, because they form agglomerates of between 1 and 5 µm that are, in many cases, interconnected with each other, would leave preferential gas paths through them with low resistance to permeation. The net effect would be a decrease in the effective thickness of the MMMs (see diagram in Figure 10), with thickness reduction being predominant compared to the decrease in permeability due to cross-linking of the polymer.

#### 3.2.3. CO_2_ and N_2_ Adsorption Measurements

As an additional method to clarify the permeability variation behavior due to the Ar plasma treatment, a series of CO_2_ and N_2_ adsorption measurements have been carried out before and after plasma treatment. The results on the pore size distributions obtained with CO_2_ are compatible with the permeability results of He, O_2_, and N_2_.

**Figure 11:**
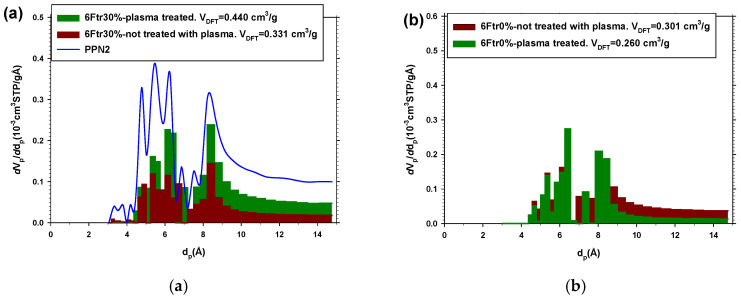
Pore size distribution obtained using NLDFT from CO_2_ adsorption isotherms at 273.15 K for pure PPN2 and for membranes with and without filler: 6Ftr30% plasma-untreated and plasma-treated (**a**); 6Ftr0% plasma-untreated and plasma-treated (**b**) (Ar plasma treatment: 29.6 W for 8.5 h).

The samples that have PPN2 show an increase in the pore population, compatible with high gas access to PPN2 due to the surface etching of the film. An example for 6Ftr30% is shown in Figure 11a, where it is seen that the plasma treatment produces an increase in the pore population throughout the range (*V*_DFT_ = 0.440 ± 0.006 cm^3^/g with plasma versus *V*_DFT_ = 0.331 ± 0.003 cm^3^/g).

On the contrary, in the case of membranes without filler, the pore population decreases when the sample is treated with plasma (*V*_DFT_ = 0.260 ± 0.003 cm^3^/g with plasma vs. *V*_DFT_ = 0.301 ± 0.003 cm^3^/g). In Figure 11b, this fact is clearer for the larger pores. This reduction in pore volume is consistent with the permeability reduction. At the same time, the fact that the plasma modification is only superficial (densification of the surface by cross-linking of the polymer chains) is compatible with both the permeability and adsorption data since, in both cases, gas penetration in the matrix is made more difficult. Along the same lines, if we evaluate the FFV for the samples before and after plasma treatment, the FFV suffers a reduction of 15.8% when the sample does not have any filler, whereas it increases by 24.8% when it has 30% of PPN2. FFV was evaluated from CO_2_-adsorbed volume, and the densities were measured via Archimedes’ principle [27].

**Figure 12:**
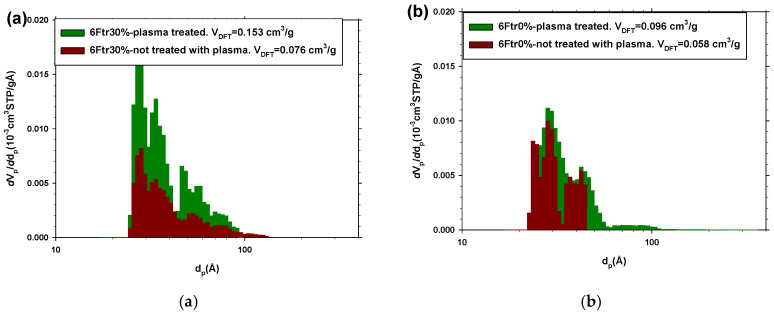
Pore size distribution via NLDFT from N_2_ isotherms (at 77 K) for samples with and without filler: 6Ftr30% plasma-treated and non-treated (**a**); 6Ftr0% plasma-treated and non-treated (**b**) (Ar plasma treatment: 29.6 W for 8.5 h).

N_2_ at 77 K has much less capacity to penetrate the polymer than CO_2_. This is due to the larger size of N_2_ and, above all, because the working temperature must drastically reduce the mobility of the polymer chains and therefore drastically reduce the diffusivity of the gas. Because of this, nitrogen adsorption isotherms provide information on the larger surface micropores that must be related to the surface roughness, due to cavities and other defects, and to the presence of accessible PPN2 on the surface. The distribution obtained by NLDFT with N_2_ isotherms shows detectable pore populations (15–150 nm) that are compatible with the proposed hypotheses. As an example, Figure 12 shows that the distribution of the samples treated with plasma has a greater population throughout the size range. This difference is more pronounced for the MMMs (Figure 12a). Furthermore, when it is a pure polymer sample, the difference is smaller in terms of frequencies, but the plasma generates larger pores (Figure 12b); that is, as we have already mentioned, the plasma increases the surface roughness and gives access to nearby PPN2 particles on the surface. The BET area measured from N_2_ isotherms corroborates the fact that plasma treatment contributes to increasing the specific porous area of the samples (see Table 8).

The hypothesis that plasma treatment increases the densification by cross-linking the polymer surface should give an increase in Tg due to the loss of mobility of the polymer chains. However, its determination is difficult because this should only occur in a very superficial layer of the film in which the plasma treatment can act. In any case, some Tg measurement tests were conducted with DSC. The results show that in both cases (in pure polymer membranes and in MMMs), Tg increases due to the plasma treatment. However, the uncertainty in the measurements is high since the increment is very small and the dispersion of the accidental error overlaps the results (see Table S3) [14].

### 3.3. SEM Characterization

One of the objectives of the SEM study is to verify that the plasma treatment only affects the surface of the membranes. To do this, cross-sectional images of the samples were taken, fractured by bending at the temperature of liquid nitrogen. It was observed that the transverse morphology is very similar for the samples treated and untreated with plasma. In fact, they show greater differences due to the presence of PPN2 or its TR samples than due to the effect of plasma (Figures S17 and S18).

**Figure 13:**
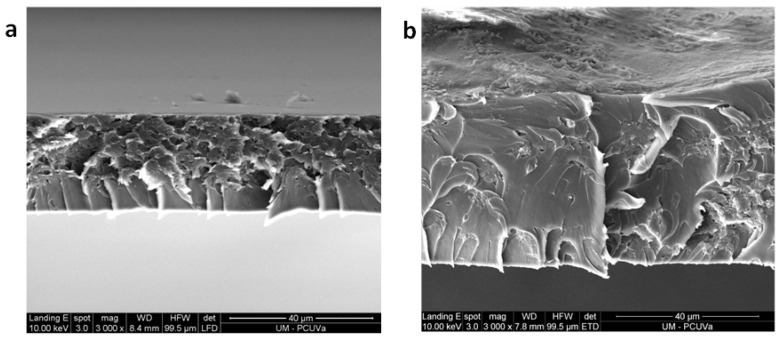
SEM images of MMM 6F20%: untreated (**a**); plasma-treated (**b**) (Ar plasma for 30 min at 10.2 W plus 12.5 h at 29.6 W).

However, SEM images of the membrane surface show significant modifications (already observed by AFM) that seem to be due to an etching process. An example is shown in Figure 13, where it is seen that the surface exposed to the plasma shows high roughness.

**Figure 14:**
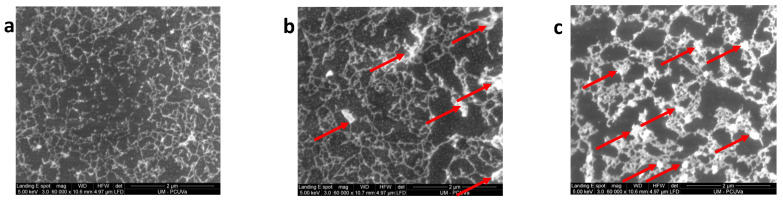
SEM surface images for plasma treated samples (Ar plasma for 30 min at 10.2 W plus 12.5 h at 29.6 W): tB0% (**a**); tB20% (**b**); 6Ftr20% (**c**). Red arrows point to some PPN2 particles.

**Figure 15:**
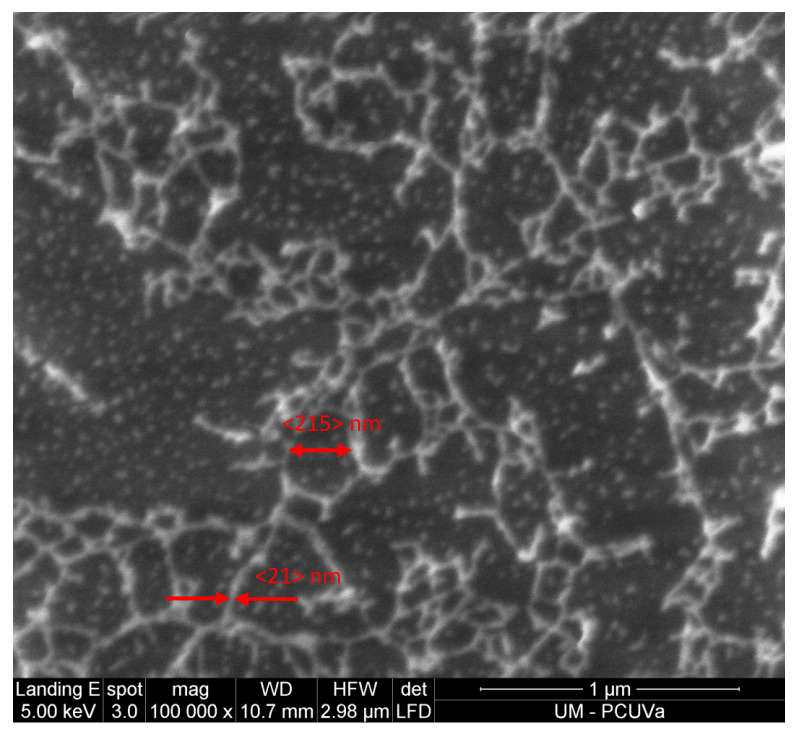
Magnification of Figure 14a.

Detailed morphological analysis of the surface allows for the analysis of this effect. In Figure 14, some examples of surface images for plasma-treated samples are shown. The topography is similar to that observed with AFM. In all cases, a surface network formed by chains of nanospheres of the polymer matrix can be seen. It can also be seen that this surface network is supported by a matrix formed by nanospheres (indicated with red arrows) of similar sizes to those of the surface network itself. In Figure 15, a magnification of Figure 14a is presented in which the typical size of the surface network cells and nanospheres, obtained via AFM for the membranes of the tB#% and tBtr#% family, has been indicated. In the images of Figure 14b,c, some PPN2 agglomerates can also be seen that are exposed after plasma treatment (indicated with red arrows). The distribution of PPN2 particles obtained from SEM images is like that obtained from AFM images.

### 3.4. Interpretation of the Structure

The results of Section 3.1 and Section 3.2 indicate that the PPN2 agglomerates are not well integrated into the matrix, mainly due to the lack of a consistent union between the particles that form it. However, the external part of the particles that form the agglomerate or the isolated PPN 2 particles are well adhered to the polymer matrix. Both SEM and AFM images corroborate this good integration between filler and matrix. Although force modulation and phase contrast techniques have not revealed the existence of a densification of the polymer matrix, it should be present. Perhaps the differences in hardness are small compared to the sensitivity of the technique, or the thickness of this layer is less than the resolution achieved under the measurement conditions.

**Figure 16:**
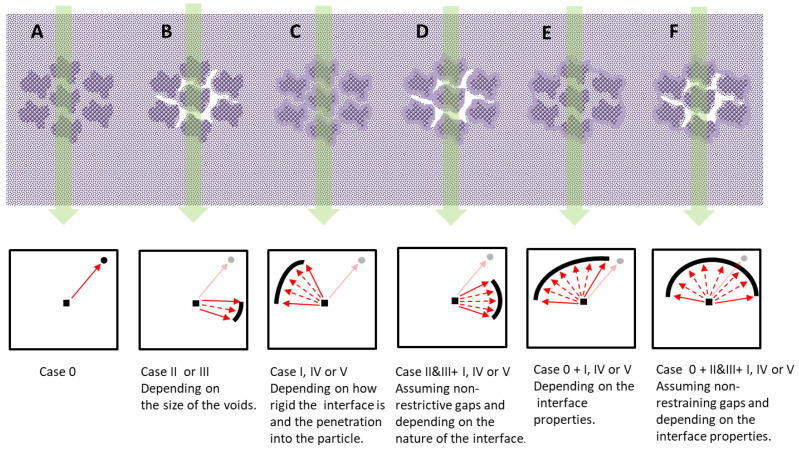
Scheme of possible structural configurations of the MMMS (**A**–**F**) of this work and their possible equivalence (correspondence marked with green arrows) with the permeation model proposed by Moore and Koros [23].

In 2005, Moore and Koros studied the possibilities of integration between the matrix and the filler in MMMs, and how this structure should impact the gas transport mechanism [21]. The study was carried out for a zeolite inside a polymer matrix, without assuming the existence of agglomerates of filler particles. However, it is easy to consider how this can be translated to systems where there is some agglomeration of filler particles. Figure 16 proposes a series of cases that we can find when we assume that the filler particles form agglomerates. Assuming the model of Moore and Koros, case 0 corresponds to perfect integration without densification of the matrix, where the filler is more permeable and selective than the matrix. Case I corresponds to the appearance of a dense layer (more selective and less permeable) covering the filler with good integration. Cases II and III assume void spaces at the interface, which allow for Knudsen diffusivity (case III is a particular case in which the thickness of the void interface is of the order of the size of the permeant gases). Cases IV and V occur when the particle of a porous filler is blocked, and this blockage reduces (case IV) or cancels (case V) the passage of gases.

Our results are compatible with schemes B, D, or F of Figure 16. And, if we assume that there is no matrix stiffening at the interfaces, we can propose scheme B. This would mean that despite the good compatibility of the polymeric matrix with the filler, there is a high tendency to build agglomerates formed by the filler particles, which makes it difficult to disperse them in the membrane manufacturing process. This could be caused by chemical interactions between parts of the different PPN2 particles that make up the agglomerate.

**Figure 17:**
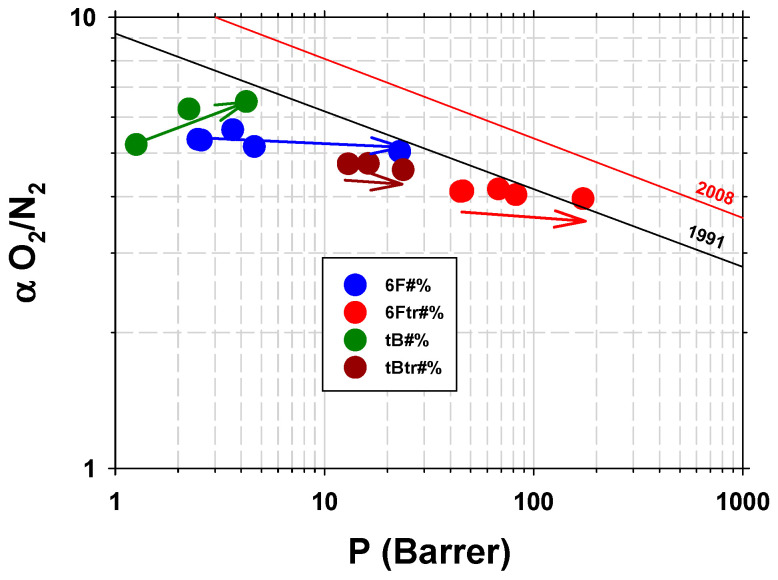
O_2_/N_2_ selectivity versus O_2_ permeability for the four families of polymers studied. The arrow marks the increase in PPN2 content from the pure polymer to the 30% of filler content.

Permeability studies previously carried out by the authors allow us to analyze the evolution of the permeability and selectivity of these families of MMMs depending on the percentage of filler [14,27]. Figure 17 shows an example for O_2_/N_2_ (a couple of gases with little interaction with the polymeric matrix). The arrow marks the increase in PPN2 content from the pure polymer to the 30% of filler content. Mostly, the gas selectivity remains constant or decreases, which, in principle, rules out a rigidification process of the matrix at the interface. This would only be compatible in the case of tB#%, but when the membrane undergoes TR, the selectivity no longer increases, which could be related to the fact that in the process of thermal rearrangement, the structure of the matrix becomes more homogeneous.

## 4. Conclusions

In this work, a large number of techniques have been used that allowed for the study of the topography and other properties of the internal structure of mixed-matrix polymeric membranes for gas separation. Especially useful have been surface abrasion and polishing, grinding with Ar plasma, and partial dissolution with appropriate solvents.

The use of AFM in lift mode complemented with force modulation and phase contrast studies has shown to be an effective tool by which to differentiate the polymer matrix from the filler polymer. This has allowed us to determine properties such as the size of the PPN2 particles and the size of the filler agglomerates.

The precise differentiation between the particles of the filler and those of the polymer matrix, when the topography does not allow for it efficiently, has been achieved by analyzing the viscoelastic properties of both materials. The FM and phase contrast images after polishing and Ar plasma etching show that there is a high affinity between the polymer matrix and the polymeric filler. However, the presence of large voids after the polishing process shows that the polymeric filler particles are difficult to disperse. Furthermore, the results of permeability (He, O_2_, N_2_) and gas adsorption (CO_2_) after s plasma attack on the MMMs are compatible with a structure in which there are agglomerates of PPN particles, with paths between them that should allow for Knudsen diffusivity.

The permselective properties and the gas adsorption study of the membranes after treatment with Ar plasma indicate that the surface of the polymer matrix became densified. This results in a decrease in permeability (He, O_2_, and N_2_) and CO_2_ adsorption capacity. However, PPN2 particles emerge to the surface. This produces preferential pathways for gas diffusion, which increase both the permeability and the CO_2_ adsorption capacity for the MMMs.

ATR-FTIR, TGA, DSC, and WAXS studies indicate that the Ar plasma modification is only relevant in the surface layer on the membrane. This has been confirmed with SEM images, which further support the morphological conclusions from the AFM results.

The structure of nanospheres that form the surface of the membranes when visualized with AFM or SEM has also been analyzed. Although these structures are common in this type of polymer materials, it has been shown that their size depends, among other possible factors, on the nature of the polymer. Furthermore, plasma treatment forms a surface network that could be related to the Debye length of the Ar plasma.

The set of techniques used in this work constitutes a package of tools that proved to be useful in the characterization of mixed-matrix membranes. An intensive use of these techniques to increase the repertoire of case studies could help improve the effective development of this type of material for advanced gas separation applications.

## Figures and Tables

**Table 1:** Nomenclature of the samples studied in this work.

Sample	Polymer	#% (%wt of PPN2 in the MMM)
6F#%	6FCl-APAF	0, 10, 15, 20, 30, 40
6Ftr#%	TR-6FCl-APAF	0, 10, 15, 20, 30, 40
tB#%	tBTmCl-APAF	0, 20, 30, 40
tBtr#%	TR-tBTmCl-APAF	20, 30, 40

**Table 2:** Densities ratios $\left(\rho_{matrix}/\rho_{filler}\right)$ and polymeric matrix density.

MMM	$\left(\rho_{matrix}/\rho_{filler}\right)$	$\rho_{matrix}$ (g/cm^3^) ^a^	$\rho_{matrix}$ (g/cm^3^) ^b^
6F#%	1.39 ± 0.06	1.38 ± 0.07	1.460 ± 0.005
6Ftr#%	1.35 ± 0.08	1.34 ± 0.08	1.435 ± 0.002
tB#%	1.20 ± 0.09	1.19 ± 0.10	1.308 ± 0.008
tBtr#%	1.16 ± 0.06	1.15 ± 0.07	1.200 ± 0.019 ^c^

^a^ This work from AFM. ^b^ Archimedes’ principle [14,27]. ^c^ Calculated from extrapolation of MMMs data. It has not been possible to obtain a TR of the pure polymer.

**Table 3:** Mean values and standard deviations for hole distributions in the case of MMMs with 10% and 15% of PPN2.

MMM	μ ± σ (μm)
6F10%	2.4 ± 1.3
6F15%	3.2 ± 1.7
6Ftr10%	2.5 ± 1.4
6Ftr15%	3.1 ± 1.9

**Table 4:** Mean values and standard deviation of the particle size distribution of PPN2 as obtained by FM-Map images (scans 1 × 1 μm and 5 × 5 μm).

MMM	Force Modulation μ ± σ (μm)
6F#%	87 ± 42
6Ftr#%	76 ± 35
tB#%	78 ± 39
tBtr#%	73 ± 41

**Table 5:** Similarities and differences compilation between analyzed samples.

	Case 1	Case 2	Figures
**SIMILARITIES**	Plasma treatment > 8 h	Plasma treatment~30 min	Figure 5f
**DIFFERENCES**	Polymer 6F	Polymer tB	Figure 6a vs. Figure 6c
	Not TR	With TR	Figure 6a vs. Figure 6b
	Without PPN2	With PPN2	Figure 6a vs. Figure 6d

**Table 6:** Mean value and standard deviation for the distributions of the equivalent diameters of nanospheres ($d_{ns}$) and the surface network cell sizes for the MMM of the two polymers and their TRs.

Sample	〈$d_{ns}$〉 ± σ (nm)	〈Cell Size〉 ± σ (nm)
6F#% and 6Ftr#%	27 ± 4	281 ± 50
tB#% and tBtr#%	21 ± 3	215 ± 29

**Table 7:** Molecular weight (*M_w_* and *M_n_*) and polydispersity value of pure hydroxypolyamides, and molecular weight of their repeating units (*M_i_*) [14,27], ${\langle N_{i}\rangle }_{ns}$, and ${\langle N\rangle }_{ns}d$ for 6FCl-APAF.

Sample	*M_w_* (Da)	*M_n_* (Da)	*M_w_*/*M_n_*	*M_i_* (Da)	〈*N_i_*〉*_ns_*	〈*N*〉*_ns_d*
6F0%	161,400	105,500	1.5	722.5	12,500 ± 1800	86 ± 15
6Ftr0%	-	100,300	-	686.4	13,000 ± 1900	89 ± 17
tB0%	104,800	58,900	1.8	704.9	5400 ± 900	65 ± 11
tBtr0%	-	55,900	-	668.8	5200 ± 900	63 ± 11

**Table 8:** BET area from N_2_ isotherms for 6Ftr0% and 6Ftr30% samples without and with plasma treatment (Ar plasma at 29.6 W for 8.5 h) and for PPN2 particles.

Sample	6Ftr30% with Plasma	6Ftr30% without Plasma	6Ftr0% with Plasma	6Ftr0% without Plasma	PPN2
*S*_BET_ (cm^3^/g)	152 ± 7	101 ± 5	115 ± 5	64 ± 3	650 ± 30

## Data Availability

Data are contained within the article and Supplementary Materials.

## Supplementary Materials

The following supporting information can be downloaded at https://www.mdpi.com/article/10.3390/polym16101397/s1, from Figure S1 to Figure S18 and from Table S1 to Table S3. References [14,27,30,31,33,41,42,43,44,45] are cited in the Supplementary Materials.
