# Supplementary material for: Morphological Study before and after Thermal Treatment of Polymer-Polymer Mixed-Matrix Membranes for Gas Separations
Source: Polymers (Basel). 2024 May 14;16(10):1397. doi: 10.3390/polym16101397 (PMC11125026; doi:10.3390/polym16101397)
Supplement: Supplementary file 1 [file polymers-16-01397-s001.zip › polymers-2916728-supplementary.pdf]

Supplementary data

Morphological Study before and after Thermal Treatment of Polymer–Polymer Mixed–Matrix Membranes for Gas Separations

Pedro Pradanos [1,2,*], Cenit Soto [1,2], Francisco Javier Carmona [1,2], Ángel E. Lozano [1,3,4], Antonio Hernández [1,2] and Laura Palacio [1,2]

1 Surfaces and Porous Materials (SMAP), Associated Research Unit to CSIC, Faculty of Science, University of Valladolid, Paseo Belén 7, 47011 Valladolid, Spain; marveliacenit.soto@uva.es (C.S.); fcojavier.carmona@uva.es (F.J.C.); lozano@ictp.csic.es (Á.E.L.); antonio.hernandez@uva.es (A.H.); laura.palacio@uva.es (L.P.)
2 Institute of Sustainable Processes (ISP), Dr. Mergelina s/n, 47011 Valladolid, Spain
3 Institute for Polymer Science and Technology (ICTP-CSIC), Juan de la Cierva 3, 28006 Madrid, Spain
4 IU CINQUIMA (Centro de Innovación en Química y Materiales Avanzados), University of Valladolid, Paseo Belén 5, 47011 Valladolid, Spain
* Correspondence: ppradanos@uva.es

1. MMMs preparation

MMMs are manufactured by mixing two polymer matrixes with a microporous polymer network, PPN2, used as a filler. In a first stage, a suspension of 10% (w/v) of PPN2 is prepared in the same solvent used to dissolve the polymer. The suspension after stirring for 2 h is sonicated for 20 min at 30% of maximum amplitude (40 cycles of 20 s sonication followed by 10 s cooling-down) with a 130 W ultrasonic probe Branson 450 Digital Sonifier (Marshall Scientific, Hampton, New Hampshire, USA) in order to maximize the dispersion of the particles without damaging the material.

The polymeric films were prepared by mixing a 10% (w/v) polymer solution (NMP in the case of 6FCl-APAF and THF for tBTmCl-APAF), with the dispersion of the filler in the appropriate proportions in each case. The membranes were cast onto a well leveled glass plate. After casting, the solvent is eliminated with a controlled heating protocol on the glass plate itself. The rest of the solvent is removed in a vacuum oven at 180ºC for 12h.

The thermal rearrangement of films (TR-MMMs) was carried out in a Carbolite Split-Tube Furnace, under a N_2 purge to maintain an inert atmosphere during the thermal process. For both families of MMMs, as well as for the pure polymers, the samples were subjected at a heating ramp at 5 °C/min up to 250 °C, maintained for 15 min, then the temperature was raised at 5 °C/min up to the target temperature (375 °C) and the samples were kept at that temperature for 15 min to guarantee the complete conversion. Afterwards, the MMMs were cooled to room temperature at 10 °C/min, in an inert atmosphere. The conversion from o-hydroxypolyimides to polybenzoxazoles was verified using FTIR. The obtained membranes presented a thickness of 40–60 μm. A more detailed description can be found in other previous works of the authors [14,27].

2.- Ar plasma treatment conditions

Table S1: Treatment times with argon plasma, with a gas flow of 0.50 cm^3/min STP at a pressure of 75 Pa. The third column shows the characterization technique used for the samples after plasma treatment. AFM stands for Atomic Force Microscopy

Time at 10.2W	Time at 29.6W	Technique used
10 min	0 min	AFM
30 min	0 min	AFM
30 min	30 min	AFM
30 min	2h (120 min)	AFM
30 min	7 h (420 min)	AFM
30 min	8 h (480 min)	AFM
30 min	12.5 h (750 min)	AFM, SEM
0 min	8.5 h (510 min)	TGA, DSC, ATR-FTIR, WAXS, thickness, CO_2 adsorption, N_2 adsorption and gas permeation.

3.- Percentage of the surface with voids and area fraction.

From the topographic images of the polished surfaces, the proportion of the surface with voids versus the flat surface was determined. Fig. S1 shows an example of this analysis for the face A of the 6F10% samples, by using the Nanoscope software. In this case when there is 10% w/w of PPN2, 80.72% of the surface is smooth, which means that the remaining 19.28% corresponds to holes produced by filler particles that have been detached after the polishing process. In the Fig. S1, the red color corresponds to the flat surface and the green one to the voids (surface below the reference level). The red oval highlights the percentage value area above this reference level.

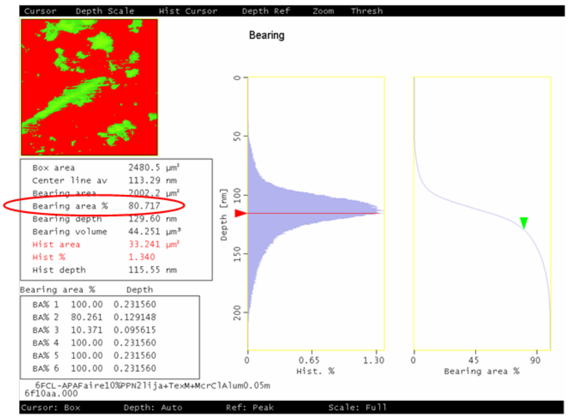

Figure S1: 6F10% with all polishing treatments: Bearing analysis of the surface occupied by PPN2 particles that have been detached from the material by the polishing process.

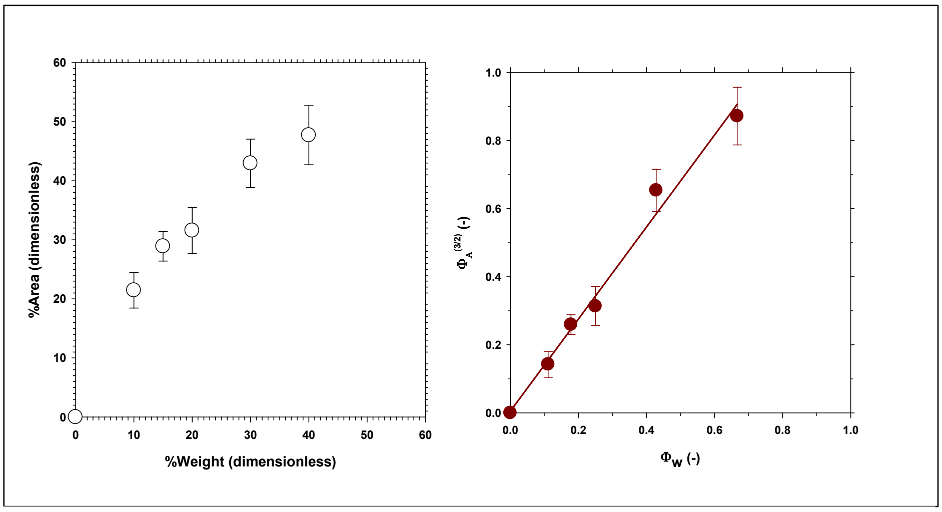

Figure S2: Percentage of surface corresponding to holes versus % w/w of PPN2 (a) and area fraction ($\Phi_A^{3/2}$) versus weight fraction (Φ_w) (b), for the series 6Ftr#%.

Fig. S2 shows the percentage of surface corresponding to holes varies versus the % w/w of PPN2 and area fraction versus weight fraction in the 6Ftr#% membrane series.

4. Holes distribution for polished surfaces.

The study of the distribution of the void spaces left by the filler agglomerates after the polishing process was carried out for membranes with 10 and 15% PPN2. This analysis was also attempted for samples with higher percentages of filler but the high population of agglomerates derived in linking of the holes of the studied surface which makes the interpretation of these distributions difficult. This study was carried out with the ImageJ [31] image analysis software by which each of the areas corresponding to the holes was determined and assimilated to circles

of size d_{circle}. Fig. S3 shows an example of the voids frequencies versus their size and the fit to a Gaussian distribution for sample 6F10%.

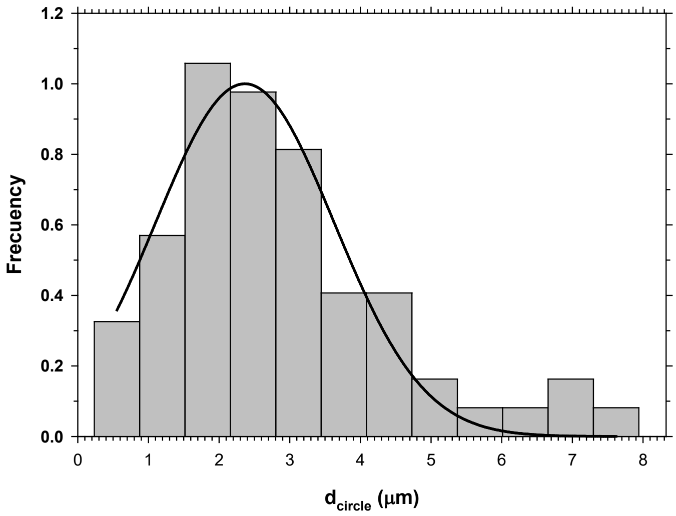

Figure S3: Holes frequency as a function of their size and the Gaussian fit for 6F10% sample.

It is observed that the size of the voids, which is the imprint that the PPN2 agglomerates have left on the surface, is around 2 μm. However, a wide distribution of sizes can be seen. For the samples studied, the data obtained for the mean value, μ, and its standard deviation, σ, correspond to the analysis of 10 images, 5 of face A and 5 of face B.

5. Roughness study for grinding and polishing samples.

The roughness was determined according to the root mean square (RMS), R_q:

$$R_q = \sqrt{\frac{1}{n}\sum_{i=0}^{n}(Z_i - Z_m)^2}$$

where Z_m is the mean value of the height of the scanned surface and Z_i is the distance to this baseline [41]. We analyze both sides of the membranes, but no significant differences were found. As expected, in all cases, it is seen that the roughness increases with the size of the swept surface [42]. For each scan size, an increase in R_q is observed with the percentage of PPN2 for the original, roughened out or polished samples and with all the polymers (see Fig. S4-a for the case of face A

in 6F#% samples, without treatment). Fig. S4b shows the case of 6F10% that has undergone the entire grinding and polishing sequence (up to polishing with 0.05 µm alumina suspension).

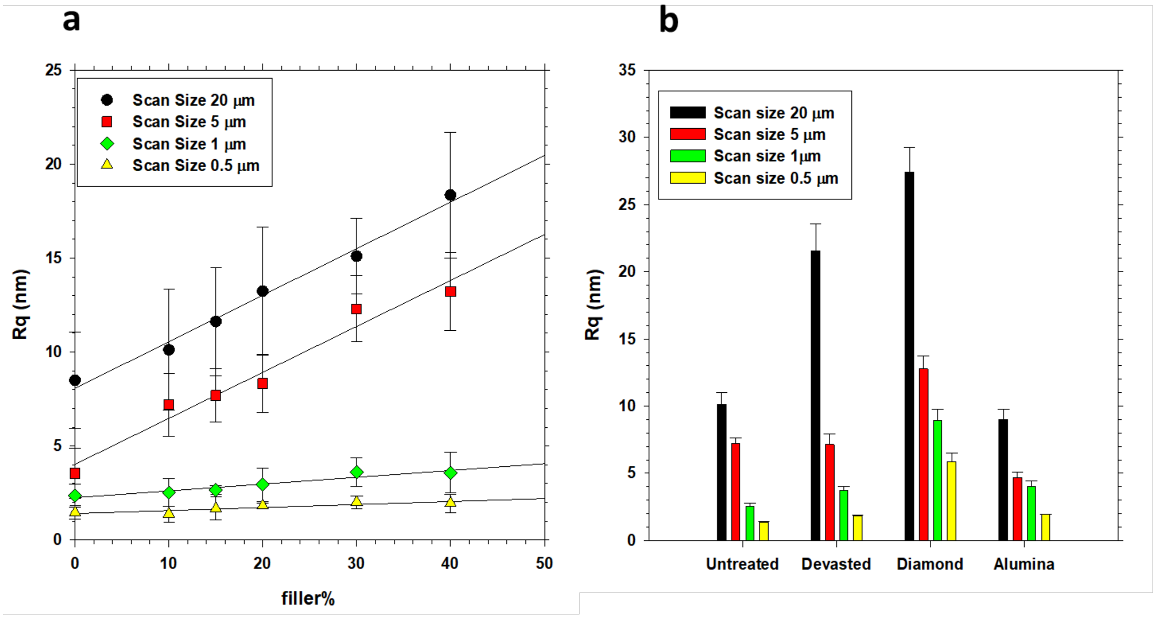

Figure S4: R_q from topographic AFM images versus filler content for untreated membrane 6F#% and for different scan sizes(a) and R_q of 6F10% MMM for different grinding and polishing treatments and different scan sizes (b).

An increase in roughness is seen with grinding and even especially with the diamond suspension polishing. However, polishing with alumina suspension gives roughness values close to that for untreated samples. This seems to indicate that diamond suspension polishing could be avoided, achieving similar results. The relative low roughness of the samples polished with alumina suspension is what has allowed this study to be carried out, since a greater roughness would have screened the holes left by the detachment of PPN2.

6. Phase contrast study for polished samples

As mentioned in the main text, the images of the surfaces after the polishing process show the presence of voids. To rule out that they could be caused by poor adhesion between PPN2 and the polymer, the phase contrast signal was analyzed, which gives information about the presence of materials with different viscoelastic properties. These images show that in the areas where holes appear, there is a change in the phase of the cantilever oscillation (Fig. S5). Even slight phase changes compatible with isolated PPN2 particles embedded in the polymer can be seen.

However, it is known that a phase change can also occur due to abrupt topographic changes [30]. In Fig. S5a we see an example where the topographic holes in the left image translate into highlights in the phase image. Increasing the magnification, Fig. S5b shows that in addition to the phase change produced by the holes, dark spots are seen in the image of phases with sizes compatible with isolated PPN2 particles. In the same way, as for the analysis of voids, the distribution of filler particles embedded in the surface of the membranes studied has been

obtained from the phase images. The parameters of the fit to a Gaussian distribution are collected in the "Phase" column of Table S2.

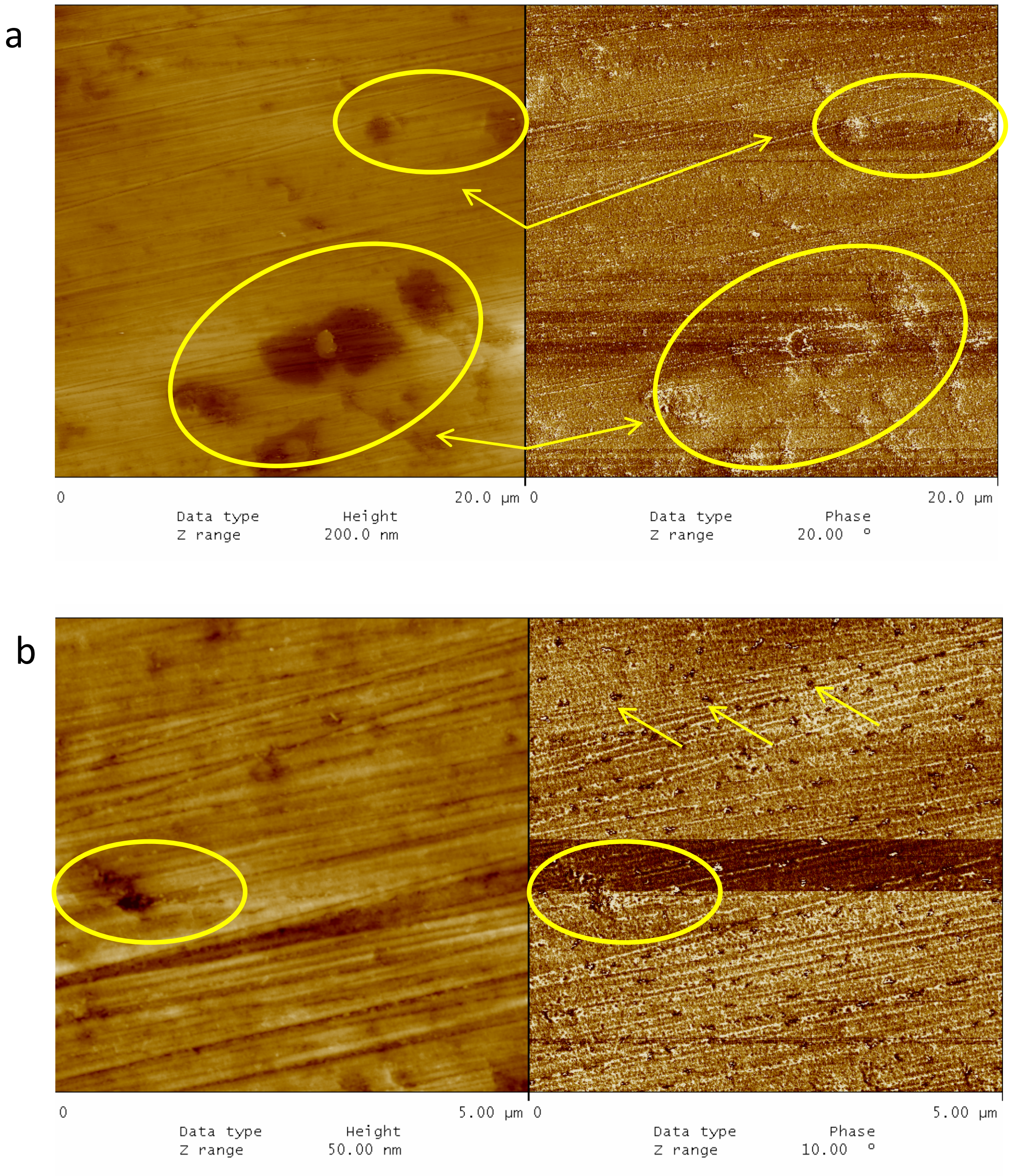

Figure S5: Topographic (left) and phase (right) images for a polished sample of 6F10%: scan size 20×20 µm (a) and 5×5 µm (b)

For the case of TR samples with polished surfaces, if they are treated with NMP, leaving a drop on the surface for 10 minutes before washing it with water, it is possible to see the filler particles more clearly. This is because NMP is not capable of dissolving either TR-polymer or PPN2, but it can clean the surface of any remains of polymer not transformed into TR and other substances adhered during polishing. Fig. S6 shows the surface image (topography and phase) for the 6Ftr10% after polishing and cleaning with NMP. Under these conditions, the distribution of PPN2 particles was measured for TR samples and the data are collected in the column "Phase+NMP" of Table S2. Each of the values in the table corresponds to at least 5 images for each MMM sample with scans of 1×1 µm and 5×5 µm. In the non-TR samples, this has not been

possible because the NMP dissolves the polymer very easily and generates highly rough surfaces that are difficult to study, from this point of view.

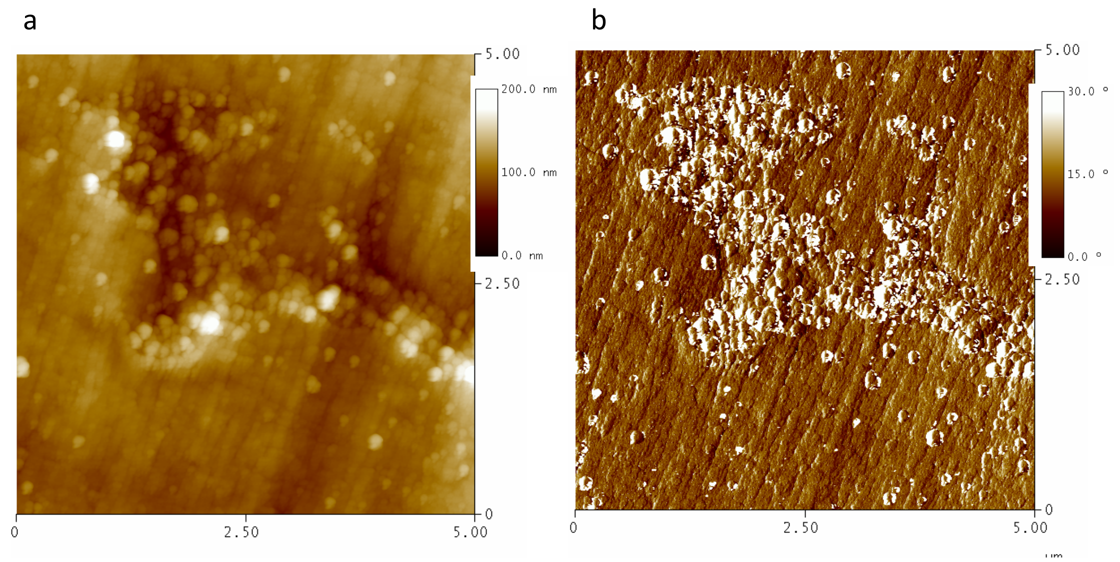

Figure S6: 6Ftr10% polished and cleaned with NMP: topography (a) and phase contrast (b)

Table S2: Mean value and standard deviation for the PPN2 particles distributions of the MMMs studied from different techniques. Data analysis was done with Image J software.

**MMM**	**Phase μ±σ (nm)**	**Phase+NMP μ±σ (nm)**	**Phase+NMP-Interleave μ±σ (nm)**
6F#%	84±31	---	---
6Ftr#%	79±29	87±50	70±38
tB#%	75±37	---	---
tBtr#%	77±33	72±42	77±40

To verify that the phase changes are due to different viscoelastic properties and not to topographic changes, the interleave scanning technique in negative lift mode has been used (see AFM description in the experimental section). An example of the topography and phase image with interleave scanning in negative lift mode is shown in Fig. S7. These images clearly show PPN2 particles that have different viscoelastic properties than the polymer matrix and that are embedded in it. The figure shows (see upper and lower areas of the images) that where there are topographic voids, a high density of PPN2 particles appears that cover the entire surface of the hole. This means that when part of the PPN2 agglomerate is lost in the polishing process, the particles of PPN2 that were in contact with the polymer are kept there. This would indicate a good adhesion between the polymer and the PPN2 particles in the MMM. At the same time, the appearance of voids is due to a low interaction between the PPN2 particles themselves that form the agglomerate. From these types of images, the distribution of particle sizes has been obtained, which is included in the "Phase+NMP-Interleave" column of Table S2.

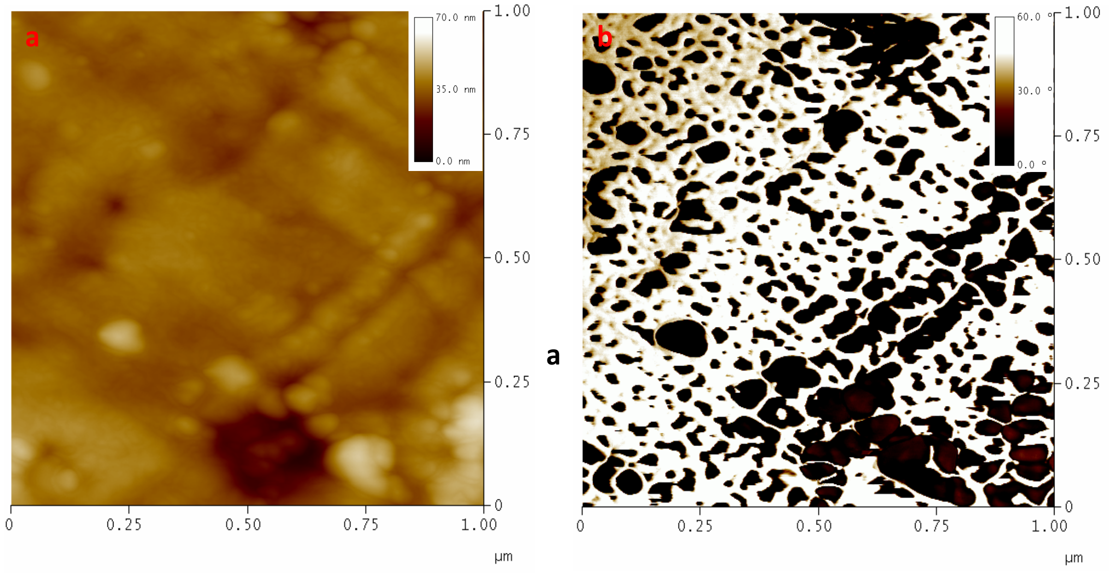

Figure S7: 6Ftr10% simple polished and cleaned with NMP, scanned with a tapping tip in its natural frequency of oscillation (296-351kHz): topography (a); phase image acquired with interleave scanning in negative lift mode (b).

7. Study by AFM of samples treated with Ar plasma.

Treatment of the samples with Ar plasma for 10 minutes at a power of 10.2W allows the removal of the contamination layer and the obtaining of AFM images of the samples surface with high resolution. Fig. S8a shows the surface dead-end pores on face A of the 6F-APAF membrane without PPN2 and no thermal rearrangement. When PPN2 is present (Fig. S8b) or the membrane has been thermally treated (Fig. S8c) these cavities do not form. The dead-end pores have not been detected on face A of the other studied polymer (Fig. S8d).

The depth of the dead-end pores or cavities of face A in 6F0% samples with the different times and/or powers of Ar plasma treatment can be determined with the profile lines of the AFM topographic images. Two examples are shown in Fig. S9, one for short treatment time and one for a long one.

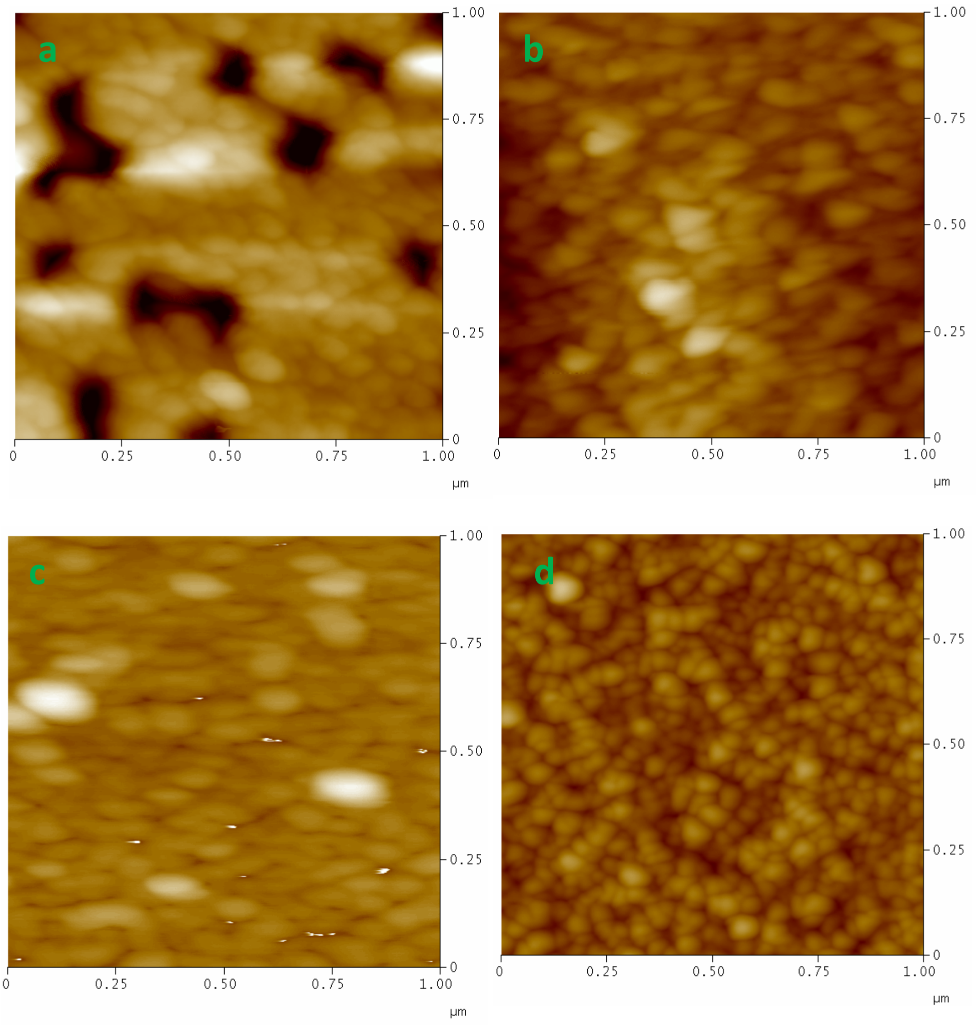

Figure S8: AFM images of surface A of membranes treated with Ar plasma for 10 minutes at 10.2W. 6F0% a), 6F20% b), 6Ftr0% c), and tB0% d).

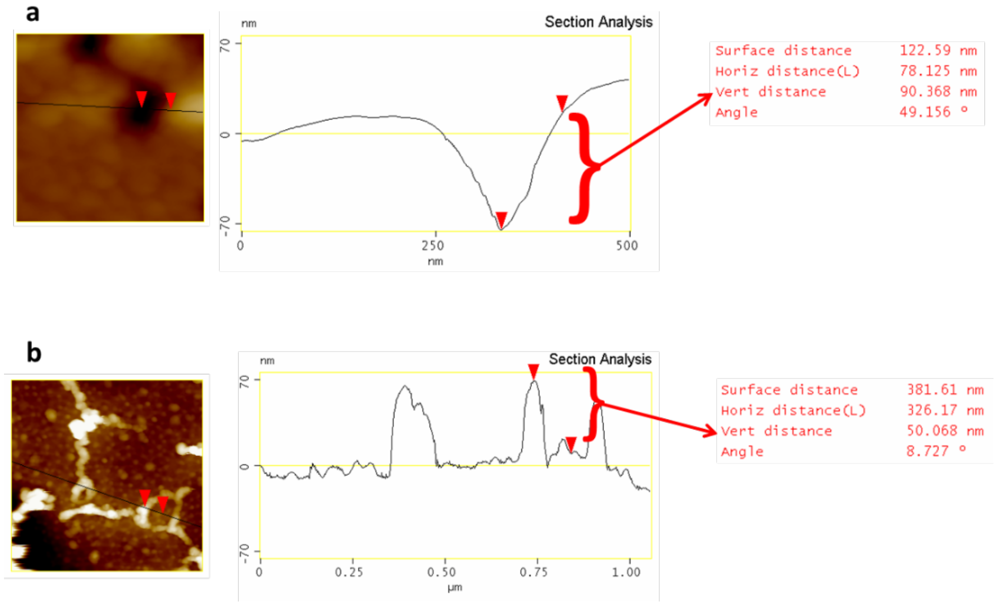

Figure S9: Profile line to analyze the depth of the dead-end pores at different Ar plasma treatment times for 6F0%: t=10 min (10.2 W) a); t=30 min (10.2W) + t=12.5 h (29.6W) b).

The roughness analysis carried out on the AFM topographic images shows that in all cases R_q increases with the Ar plasma treatment. The increase is very fast at first and tends to stabilize for times greater than 3 hours. An example for 1×1 μm image sizes is shown in Fig S10. The behavior is similar for other scan sizes. However, the absolute values of R_q increase with scanning area in the same way as in Fig. S4. At the same time, Fig. S10 clearly shows how the presence of PPN2 produces a significant increase in roughness throughout the treatment times.

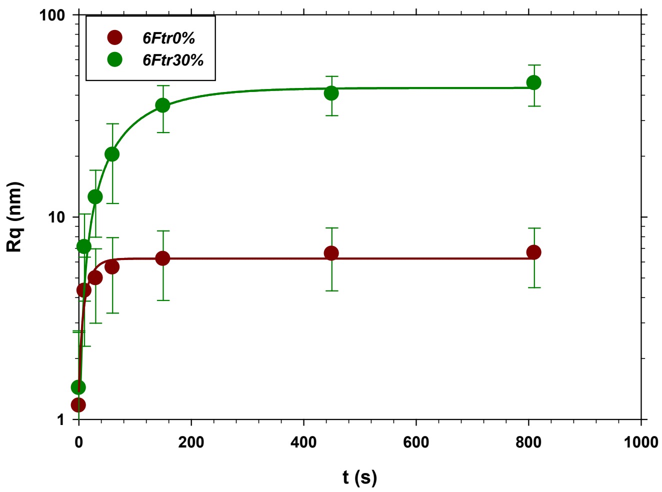

Figure S10: Rq as a function of treatment time with Ar plasma (power 29.6W) for the samples 6Ftr0% and 6Ftr30%.

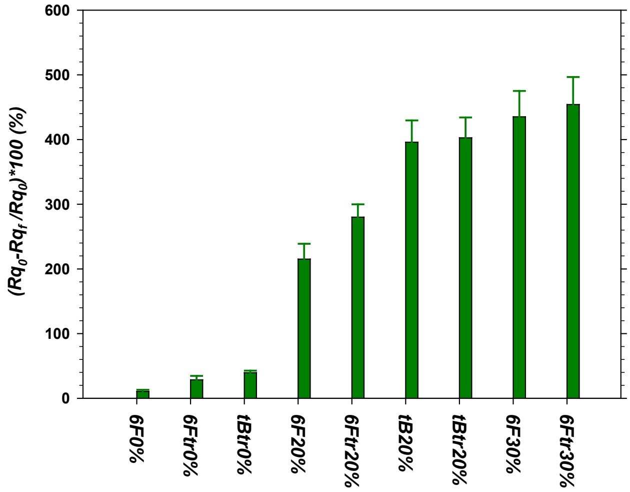

Figure S11: Increase in roughness in % due to Ar plasma treatment: for different samples: increment of Rq_f (power 29.6 W during 12.5 h) with respect to Rq_0, (power 10.2 W for 10 min).

The increase in roughness with plasma treatment depends on the type of polymer, the TR process, the presence of PPN2 and its concentration. Examples of this behaviour are shown in Fig. S11. It is clearly seen that in pure polymers the increase is much smaller than in MMMs. And in MMM samples the roughness grows with the PPN2 content. It is also observed that, in all cases, polymers and MMMs that have undergone thermal rearrangement increase their roughness more than those that have not. Furthermore, the polymers of the tB#% and tBtr#% families have slightly higher increments than those corresponding to the 6F#% and 6Ftr#% families.

Size distributions for nanospheres and surface network cells were obtained from the topographic and phase contrast images of the samples treated with Ar plasma, for long times. Fig. S12a and 12b show an example of each of the polymers. The statistical parameters have been obtained by analyzing 5 samples of each AFM image, by using the Image J software. Since all samples with a 6FCl-APAF polymeric base and their TR membranes show very similar distributions, their results have been unified into a single distribution. The same occurs with the tBTmCl-APAF polymer samples and their TRs. The distribution of nanospheres sizes is shown in Fig. S12b and 12e. The characteristic values of the distribution (mean value and standard deviation) are shown in each figure and in Table 5 of the main manuscript. The cell size distribution is shown in Fig. 12c and 12f, including the mean value and standard deviation in each figure and also in Table 5. In Fig. S12a (for 6F0%) and Fig. S12d (for tBTmCl-APAF), examples of the nanosphera size and of the cell size are marked with red arrows, to which we have assigned the mean value of the distribution, in both, topographic and phase images.

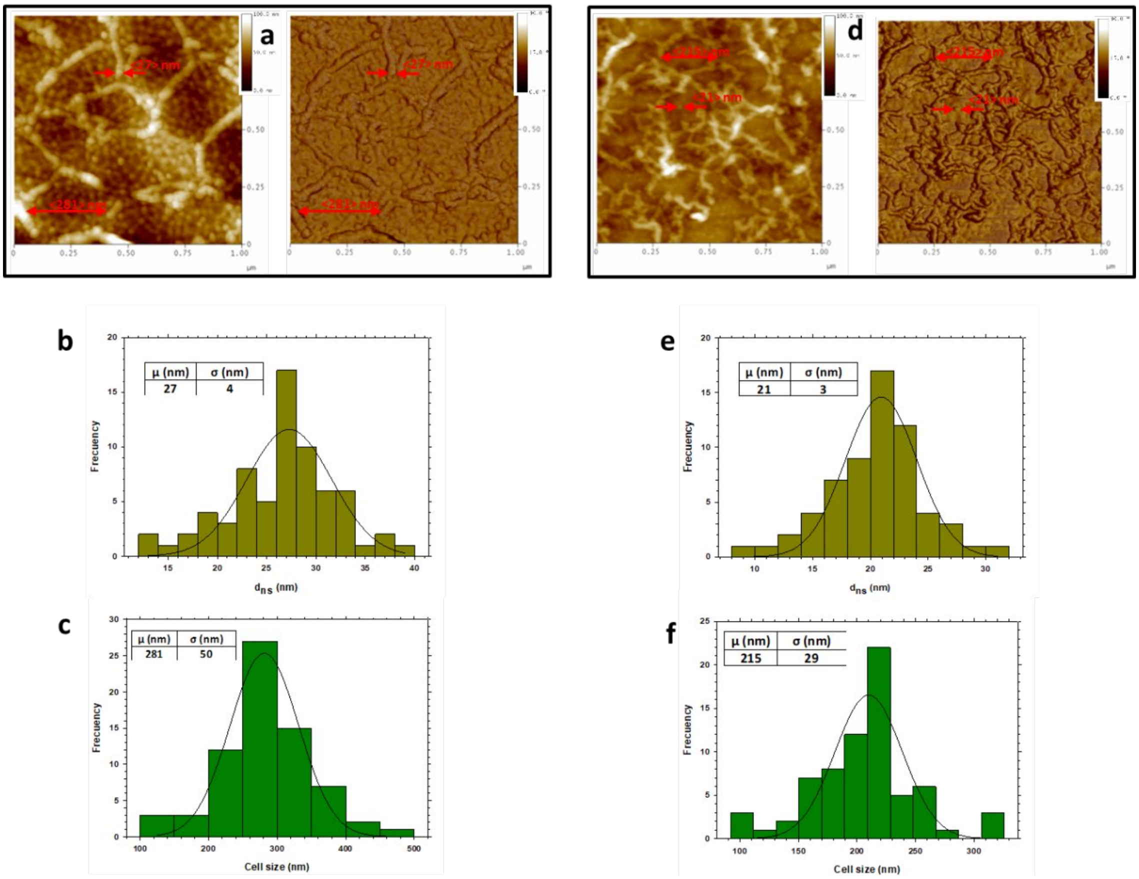

Figure S12: Membranes treated with Ar plasma for 30 min at 10.2W plus 12.5 h at 29.6W: topographic (left) and phase contrast (right) images for a 6F0% sample a); nanosphere size distribution for samples with 6FCl-APAF polymers and their TRs b); cell size distribution for samples with 6FCl-APAF polymers and their TRs c); topographic (left) and phase contrast (right) images for a tB0% sample d); nanospheres size distribution for samples with tBTmCl-APAF polymers and their TRs e); cell size distribution for samples with tBTmCl-APAF polymers and their TRs f).

To determine if the network superimposed on the surface has different viscoelastic properties than the matrix that supports it, phase contrast measurements with interleave in lift mode have been carried out. Two examples of this are shown in Fig. S13, where the technique used to remove topographic interferences (images on the left of the figure) still reveals the

presence of this network (phase contrast image on the right). This indicates that the viscoelastic properties of the network are different from the polymeric base on which they are supported.

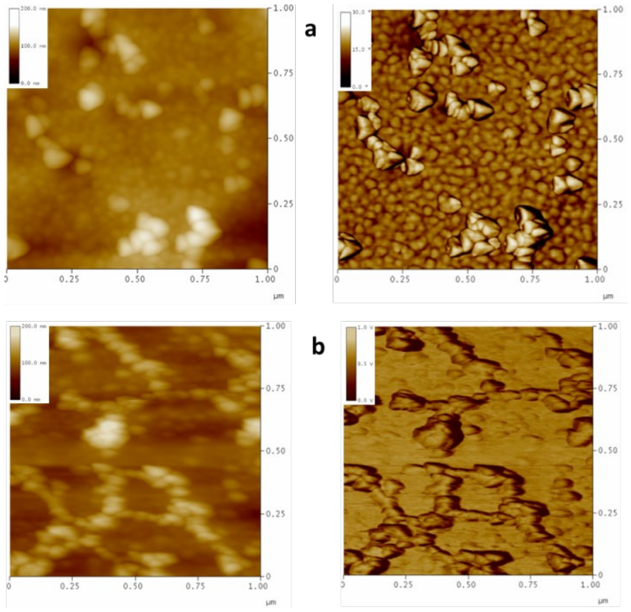

Figure S13: Membranes treated with Ar plasma for 30 min at 10.2W plus 12.5 h at 29.6W. Images of topography (left) and phase contrast with interleave in lift mode (right) for a 6F0% (a) and for 6F10% in an area where there is no PPN2 (b)

8. Study by FTIR-ATR, TGA and DSC of membranes treated with Ar plasma.

Regarding the results of the FTIR analysis, there aren't any differences concerning the position of the peaks in none of the cases studied. However, the signal intensity is lower in all cases when the membrane has been treated with plasma. This may indicate that the plasma removes part of the functional groups from the surface and/or that the possible cross-linking of the polymer chains (densification) reduces the signal intensity. Fig. S14 shows an example with two pure polymers and one of them with 20% PPN2.

TGA studies do not show differences before and after plasma treatment in any of the samples studied. This indicates that the possible processes that plasma can trigger, such as the modification of functional groups and the cross-linking of polymer chains, either do not occur, or they are not manifested in TGA thermograms. Given that in the treatment with low pressure plasma the modification is limited to a superficial layer of small thickness [13-45], without altering the bulk properties of the polymer, it is expected that these modifications will not manifest themselves in the thermogram. An example is shown in Fig. S15, where samples were heated from 50 to 1000 °C at 10 °C/min in a N_2 atmosphere.

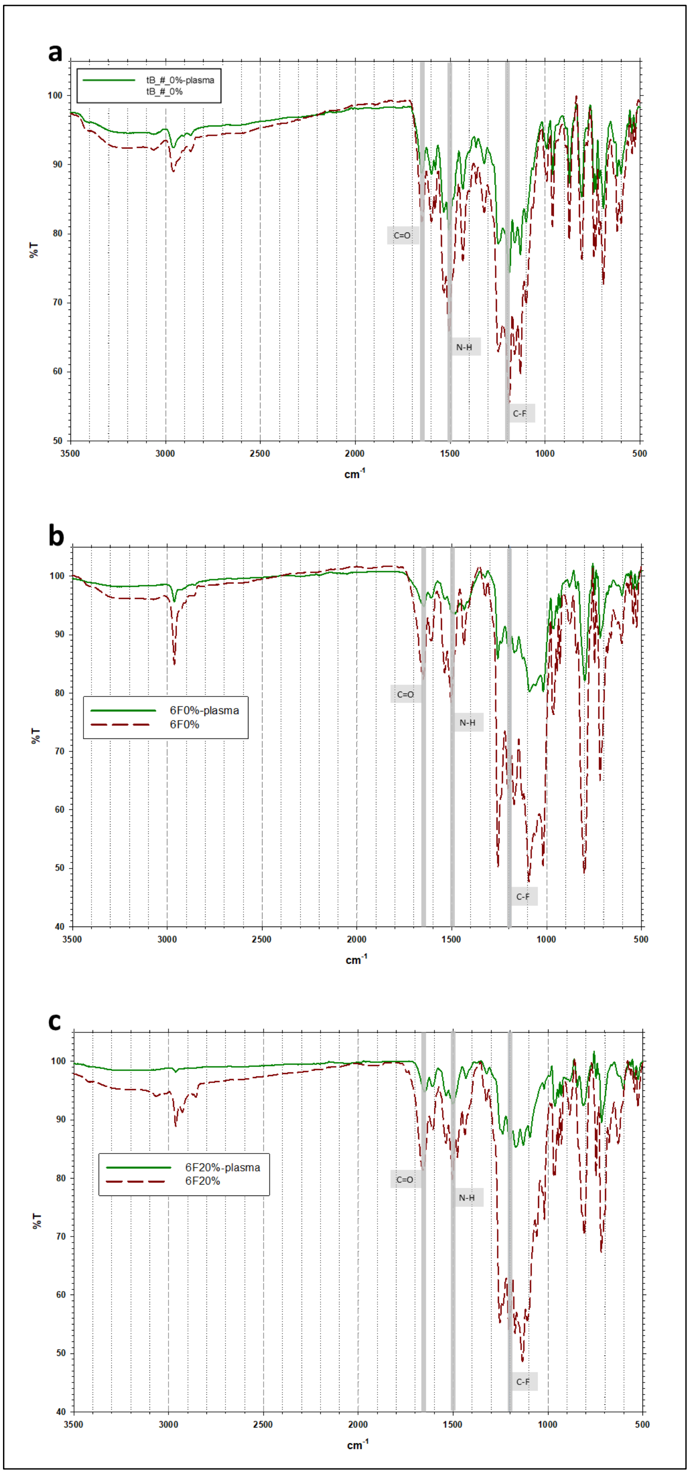

Figure S14: FTIR-ATR spectra of membranes before and after plasma treatmente (Ar plasma for 8.5 h at 29.6W). tB0% a), 6F0% b), 6F20% c).

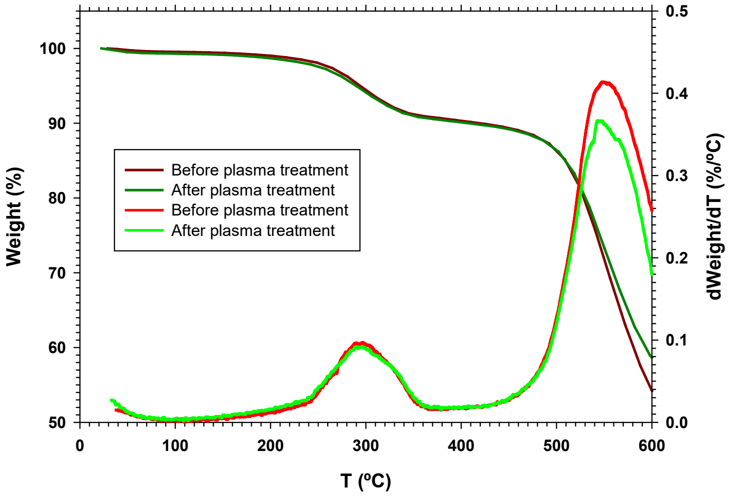

Fig. S15: TGA thermogram of a 6F10% sample beftckore and after plasma treatment (Ar plasma for 8.5 h at 29.6W).

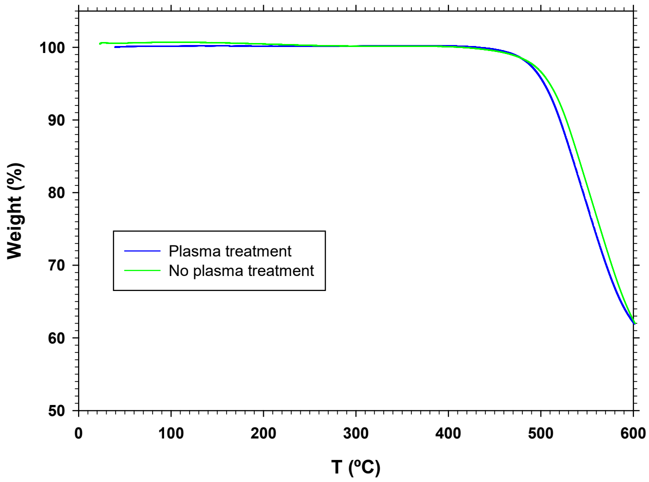

Fig. S16: TGA thermogram of two 6Ftr0% TR samples, obtained from the original HPA polymer without plasma treatment (green) and with plasma treatment (Blue) (Ar plasma for 8.5 h at 29.6W).

To analyze whether the cross-linking process of polymer chains due to plasma hinders the thermal rearrangement process, a study with TGA has been carried out. Fig. S16 shows an example where it is seen that there are no important differences in the thermogram of a TR when, before its thermal rearrangement, it has been subjected to treatment with Ar plasma.

The Table S3 shows the results of T_g (from DSC) for the same samples studied by gas adsorption.

Table S3: T_g obtained by DSC measurements for 6Ftr0% and 6Ftr30% before and after plasma treatement (Ar plasmat at 29.6W during 8.5h).

	**T_g (°C) before plasma tr.**	**T_g (°C) after plasma tr.**
6Ftr0%	325.8±0.5	326.5±0.5
6Ftr30%	330.2±0.5	330.4±0.5

9. Scanning Electron Microscopy (SEM)

SEM images of cross-sections of the membranes, fractured at the liquid nitrogen temperature, show that the plasma treatment does not modify the internal morphology of the membranes. Fig S17 shows an example of a cross-section of a MMM not treated (Fig. S17a and 17c) and treated (Fig. S17b and 17d) with Ar plasma. Only the membrane surface shows differences (Fig. 17a vs. Fig. 17b) due to the outcropping of PPN2 particles in the plasma treatment process.

The morphological differences that appear in the images of the cross sections of these membranes are fundamentally due to the presence of PPN2 and the TR process, as can be seen in Fig. S18. This was previously observed in samples not treated with plasma [33], so this morphological change is also not affected by plasma treatment.

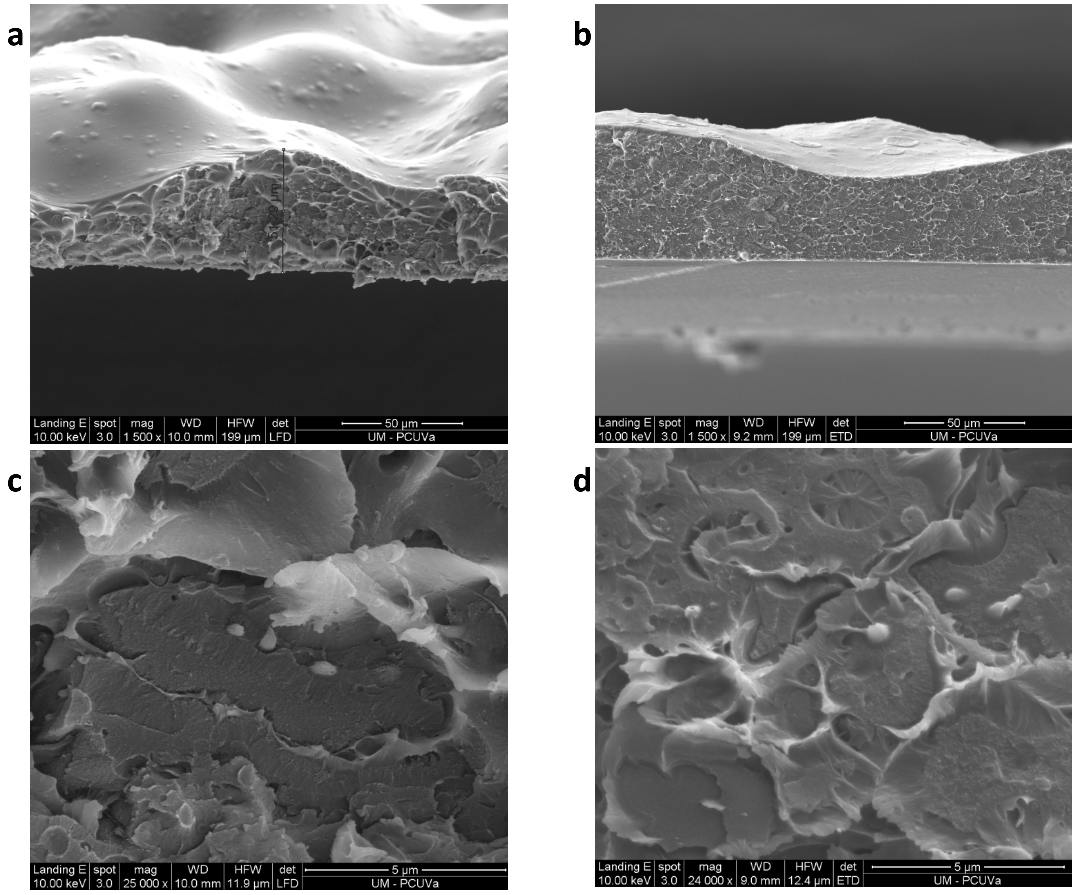

Figure S17: Transversal cross section of 6F30% sample without plasma treatment (a and c) and with plasma treatment (b and d) (Ar plasma, 30 min at 10.2W plus 12.5 h at 29.6W).

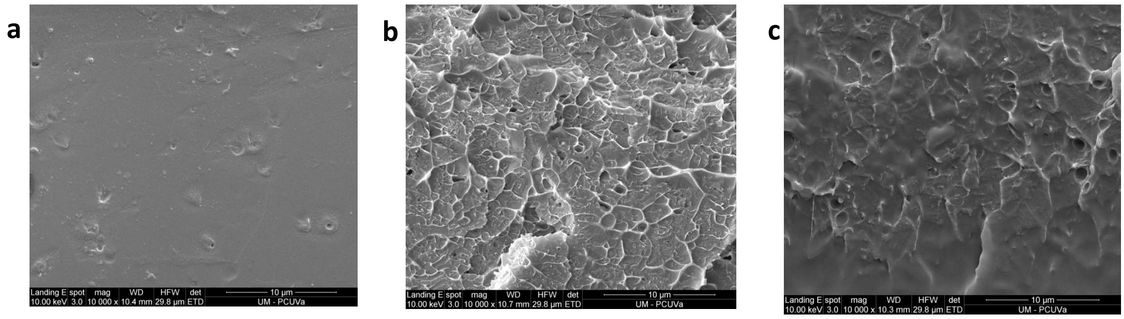

Figure S18: Cross section of tB0% (a); tB20% (b); tBtr20% (c) treated with Ar plasma for 30 min at 10.2W plus 12.5 h at 29.6W.
